# STING agonism reprograms tumor-associated macrophages and overcomes resistance to PARP inhibition in BRCA1-deficient models of breast cancer

**DOI:** 10.1038/s41467-022-30568-1

**Published:** 2022-05-31

**Authors:** Qiwei Wang, Johann S. Bergholz, Liya Ding, Ziying Lin, Sheheryar K. Kabraji, Melissa E. Hughes, Xiadi He, Shaozhen Xie, Tao Jiang, Weihua Wang, Jason J. Zoeller, Hye-Jung Kim, Thomas M. Roberts, Panagiotis A. Konstantinopoulos, Ursula A. Matulonis, Deborah A. Dillon, Eric P. Winer, Nancy U. Lin, Jean J. Zhao

**Affiliations:** 1 Department of Cancer Biology, Dana-Farber Cancer Institute, Boston, MA USA; 2 Department of Biological Chemistry and Molecular Pharmacology, Harvard Medical School, Boston, MA USA; 3 Broad Institute of Harvard and MIT, Cambridge, MA USA; 4 Department of Medicine, Harvard Medical School, Boston, MA USA; 5 Department of Respiratory and Critical Care Medicine, The First Affiliated Hospital of Sun Yat-sen University, Guangzhou, Guangdong China; 6 Department of Medical Oncology, Dana-Farber Cancer Institute, Boston, MA USA; 7 Laboratory of Systems Pharmacology, Harvard Medical School, Boston, MA USA; 8 Department of Cell Biology and Ludwig Center at Harvard, Harvard Medical School, Boston, MA USA; 9 Department of Cancer Immunology and Virology, Dana-Farber Cancer Institute, Boston, MA USA; 10 Department of Pathology, Brigham and Women’s Hospital, Boston, MA USA

**Keywords:** Tumour immunology, Cancer immunotherapy, Breast cancer, Cancer immunotherapy

## Abstract

PARP inhibitors (PARPi) have drastically changed the treatment landscape of advanced ovarian tumors with *BRCA* mutations. However, the impact of this class of inhibitors in patients with advanced *BRCA*-mutant breast cancer is relatively modest. Using a syngeneic genetically-engineered mouse model of breast tumor driven by *Brca1* deficiency, we show that tumor-associated macrophages (TAMs) blunt PARPi efficacy both in vivo and in vitro. Mechanistically, BRCA1-deficient breast tumor cells induce pro-tumor polarization of TAMs, which in turn suppress PARPi-elicited DNA damage in tumor cells, leading to reduced production of dsDNA fragments and synthetic lethality, hence impairing STING-dependent anti-tumor immunity. STING agonists reprogram M2-like pro-tumor macrophages into an M1-like anti-tumor state in a macrophage STING-dependent manner. Systemic administration of a STING agonist breaches multiple layers of tumor cell-mediated suppression of immune cells, and synergizes with PARPi to suppress tumor growth. The therapeutic benefits of this combination require host STING and are mediated by a type I IFN response and CD8^+^ T cells, but do not rely on tumor cell-intrinsic STING. Our data illustrate the importance of targeting innate immune suppression to facilitate PARPi-mediated engagement of anti-tumor immunity in breast cancer.

## Abstract (web summary)

PARP inhibitor (PARPi) therapy has demonstrated only modest efficacy in advanced breast cancer with BRCA mutations. Here the authors show that, by suppressing PARPi-triggered DNA damage and reducing dsDNA production in BRCA1-deficient breast tumor cells, tumor associated macrophages contribute to PARPi resistance, that can be overcome by STING agonism.

## Introduction

Homologous recombination (HR) deficiency confers exquisite sensitivity to poly (ADP-ribose) polymerase (PARP) inhibitors (PARPi), which have been therapeutically exploited in both ovarian and breast tumors carrying loss-of-function mutations in HR pathway genes, most commonly *BRCA1* and *BRCA2*^1^. Based on a substantial progression-free survival (PFS) benefit, three PARPi have gained FDA approval for *BRCA*-mutated ovarian cancer in both adjuvant and metastatic settings^2–5^. Moreover, maintenance treatment with olaparib was shown to confer unprecedented overall survival benefit for patients with *BRCA*-mutated relapsed ovarian cancer^6^. Compared to *BRCA*-mutated ovarian cancer, however, PARPi therapy appears to be less effective in *BRCA*-mutated breast cancer. Nevertheless, the FDA has approved two PARPi—olaparib and talazoparib—as monotherapy for *BRCA*-mutated and HER2-negative breast cancers^7–9^. Although the OlympiAD and EMBRACA trials demonstrated that PARPi significantly improved PFS, these trials found no overall survival benefit for both olaparib and talazoparib in patients with advanced breast cancer carrying germline *BRCA* pathogenic variants^10,11^, highlighting the need to understand the mechanisms of PARPi resistance in advanced breast cancers in the effort to develop strategies to improve responses to PARPi.

Our understanding of the mechanisms underlying the therapeutic efficacy of PARPi is still evolving. Since first described in 2005^12,13^, PARPi have been shown to exert synthetic lethality in HR-deficient tumor cells via multiple mechanisms, including inhibiting base excision repair (BER), trapping of PARP–DNA complexes, activating error-prone non-homologous end joining (NHEJ), and interfering with PARP1/POLQ-mediated alternative end joining (alt-EJ)^14,15^. Recently, we and others demonstrated that the immune response triggered by PARPi is also required for tumor elimination in vivo^16–18^. Using a genetically engineered mouse model (GEMM) of *Brca1*-deficient ovarian cancer, we showed that treatment with PARPi leads to release of double-stranded DNA (dsDNA) fragments by tumor cells, which activate stimulator of interferon genes (STING) signaling in intratumoral dendritic cells (DCs), thus triggering a type I interferon (IFN) response and subsequent induction of anti-tumor CD8^+^ T cells^16^. Activation of the STING pathway occurs through production of cyclic dinucleotides by cyclic GMP-AMP Synthase (cGAS), which acts as a sensor for dsDNA fragments^19^. In addition to stimulating immune cell activation, recent studies have also revealed the capacity of PARPi to activate STING-dependent tumor cell-intrinsic immunity^17,18^. The relative contribution of STING activation in immune cells and tumor cells to cancer treatment remains unclear.

Notably, clinical outcome in breast cancer is strongly affected by the tumor immune microenvironment (TIME)^20–22^. Multiple studies have shown that advanced breast tumors often exhibit a pre-existing immunosuppressive TIME characterized by significantly reduced tumor-infiltrating lymphocytes (TILs) and increased expression of immune-suppressive genes, which correlates with reduced response to chemotherapy and immunotherapy^22–24^. While increasing evidence has indicated the immunomodulatory properties of PARPi in vivo, it is currently unclear whether an immune-suppressive TIME in *BRCA*-mutant breast cancer may influence the efficacy of PARPi.

Tumor-associated macrophages (TAMs) constitute one of the most abundant and diverse immune populations in solid tumors. Although TAM phenotypes and functions are highly plastic, they can be broadly classified as anti-tumorigenic (M1-polarized) or pro-tumorigenic (M2-polarized) based on their ability to either promote or suppress tumor growth^25^. M2-polarized TAMs can exert immune suppression via multiple mechanisms, including recruitment of immunosuppressive immune cells, such as regulatory T cells (Tregs) and direct inhibition of immune effector cells such as natural killer (NK) cells and cytotoxic T cells^26^. Of note, multiple clinical studies have shown that high TAM infiltration associates with poor prognosis in the majority of cancer types^27^.

In this study, we demonstrate that the response of BRCA1-deficient breast tumors to PARPi is strongly limited by pro-tumorigenic TAMs, which not only inhibit CD8^+^ T cells but also suppress PARPi-triggered tumor cell DNA damage, resulting in reduced cytosolic dsDNA fragments and synthetic lethality, thereby dampening the activation of DNA sensing STING pathway. Addition of an exogenous STING agonist shifts TAMs from a pro-tumorigenic M2-like macrophage phenotype to an anti-tumor M1-like state and restores the synthetic lethal response to PARPi. Consequently, systemic delivery of a STING agonist combined with PARPi elicits robust anti-tumor immunity and demonstrates significant therapeutic efficacy in *Brca1*-deficient mouse models of breast cancer regardless of tumor cell-intrinsic STING expression. Our findings reveal an approach to improve the response of *BRCA1*-mutant breast cancers to PARPi therapy.

## Results

### *Brca1*-deficient breast tumors show modest response to olaparib in vivo

**Fig. 1:**
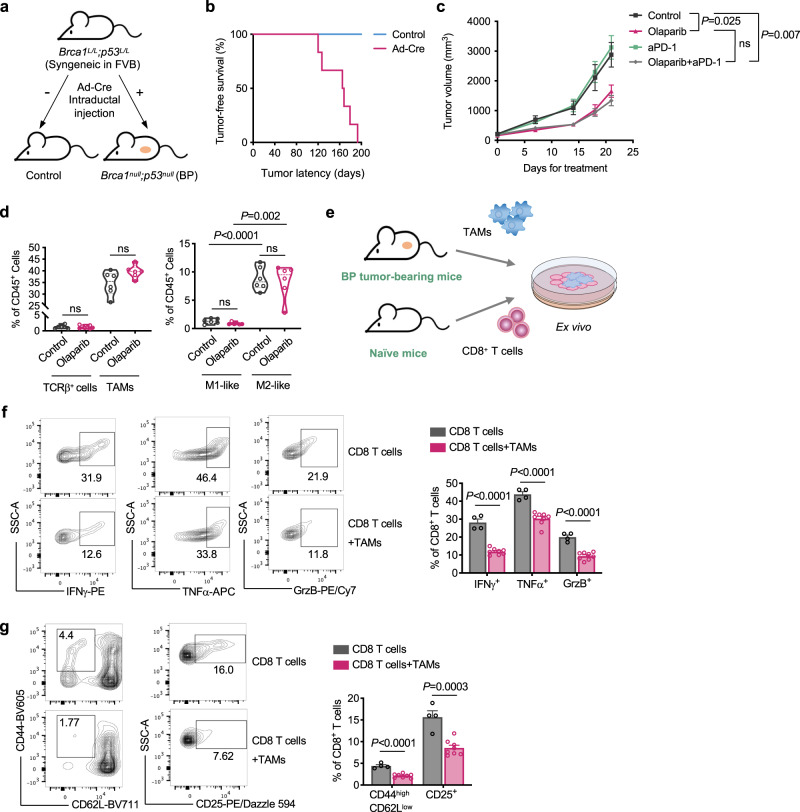
*Brca1*-deficient breast tumors have a modest response to olaparib in vivo with immune-suppressive TAMs. **a** Generation of a syngeneic GEMM of *Brca1*-deficient breast tumors by intraductal injection of adenovirus expressing Cre recombinase (Ad-Cre) directly into the lumen of mammary glands. **b** Tumor-free survival of *Brca1*^*L/L*^ *Trp53*^*L/L*^ mice with or without intraductal injection of Ad-Cre (*n* = 6). **c** Tumor growth of *Brca1*^*−/*−^ *Trp53*^*−/*−^ (BP) allografts in FVB mice treated with olaparib or anti-PD-1 as monotherapy or in combination. Control, *n* = 8; anti-PD-1, *n* = 8; olaparib, *n* = 6; olaparib + anti-PD-1, *n* = 10. **d** Flow cytometry analysis of TCRβ^+^ T cells and tumor-associated macrophages (TAMs; CD45^+^ CD11b^+^ F4/80^+^) from BP tumors in FVB mice after 21 days of olaparib treatment. TCRβ^+^ (control), *n* = 6; TCRβ^+^ (olaparib), *n* = 8; TAMs (control), *n* = 6; TAMs (olaparib), *n* = 6. TAMs were further analyzed to identify M1-like (MHC-II^high^ CD206^−^) and M2-like (MHC-II^low^ CD206^+^) polarization phenotypes (*n* = 6 for each group). Each dot represents data from a single tumor. **e** Diagram of workflow for (**f**) and (**g**). TAMs (7AAD^−^ CD45^+^ CD11b^+^ F4/80^+^) were sorted from BP breast tumors and co-cultured with splenic CD8^+^ T cells isolated from naïve mice for 2 days. **f** Analysis of cytokine production by CD8^+^ T cells co-cultured with TAMs (CD8^+^ T cells, *n* = 4; CD8^+^ T cells+TAMs, *n* = 8). **g** Analysis of the proportion of effector cells (CD44^high^ CD62L^low^) and surface expression of CD25 of CD8^+^ T cells co-cultured with TAMs (CD8^+^ T cells, *n* = 4; CD8^+^ T cells+TAMs, *n* = 8). Flow cytometry plot axes are displayed in logarithmic scale (**f**) and (**g**). Data are presented as mean ± SEM (**c**), (**f**), and (**g**), or median with quartiles (violin plots, **d**). Two-way analysis of variance (ANOVA) (**c**). Two-tailed unpaired *t* test or Mann–Whitney test (**d**), (**f**), and (**g**). ns not significant. Source data are provided as a Source Data file.

To investigate the response to PARPi in *Brca1*-deficient breast cancer, we developed a syngeneic GEMM driven by concurrent ablation of *Brca1* and *Trp53* (referred as BP), as protein-truncating *TP53* mutations are frequently found in *BRCA1*-deficient breast cancers^28^. This model was generated through intraductal injection of adenovirus expressing Cre recombinase into mammary ducts of FVB/N females carrying homozygously floxed alleles of *Brca1* and *Trp53* (*Brca1*^*L/L*^*; Trp53*^*L/L*^, Fig. 1a and Supplementary Fig. 1a). These mice developed mammary tumors around 4–7 months (median latency of 167 days) with 100% penetration (Fig. 1b). Using normal mouse mammary tissue from FVB mice as controls, we confirmed that BP tumors are negative for BRCA1 and ERα by immunohistochemistry (IHC) staining (Supplementary Fig. 1b, c). Of note, the majority of BP tumor cells displayed positive nuclear staining for Ki67 (Supplementary Fig. 1c), and histology analysis revealed that BP tumors were poorly differentiated adenocarcinomas (Supplementary Fig. 1d), consistent with that of advanced *BRCA1*-deficient breast cancer in the clinic^29^.

Notably, primary tumor cells derived from BP tumors can be cultured in vitro as well as allografted back into the mammary fat pads of syngeneic FVB/N mice, allowing detailed studies of tumor cell-intrinsic activities, as well as their interactions with the host immune system and their responses to therapeutic interventions.

We first assessed BP cell-intrinsic response to PARP inhibition in vitro. BP cells with reconstituted wild-type (WT) BRCA1 (BP + BRCA1) were used as a control. We found that olaparib treatment had little effect on BP + BRCA1 cells, but significantly inhibited clonogenic growth of BP cells in a dose-dependent manner (Supplementary Fig. 1e–g). Despite the sensitivity to olaparib in vitro, when orthotopic BP tumor-bearing FVB mice were treated with olaparib, tumors exhibited an initial slower growth than control tumors but nevertheless progressed through treatment and presented growth rates comparable to control tumors at later time points (Fig. 1c). While there was a statistically significant reduction in tumor size compared to control tumors, the effect of olaparib on *Brca1*-deficient breast tumors was modest, in contrast to its remarkable effect on a comparable mouse model of high-grade serous ovarian cancer (HGSOC) driven by concurrent ablation of *Brca1* and *Trp53* and overexpression of cMyc (referred as PBM), where we found that the ovarian tumors had a dramatic response to olaparib with a robust activation of anti-tumor CD8^+^ T cell response in the TIME, which was essential for the therapeutic efficacy of PARPi^16^.

### BRCA1-deficient breast tumor-associated macrophages mediate immune suppression

To better understand the TIME of BP tumors, we performed tissue-based multiplexed cyclic immunofluorescence (CyCIF) analysis. We found that BP tumors are highly infiltrated by CD45^+^ immune cells, of which myeloid cells (CD11b^+^, F4/80^+^ or CD11c^+^), such as F4/80^+^ macrophages, are more abundant than T lymphocytes (CD4^+^ or CD8^+^) (Supplementary Fig. 1h). We then assessed the TIME of BP tumors following PARPi treatment. Analysis of TILs revealed that CD8^+^ T cells and effector CD8^+^ T cells were not significantly changed after 7 days of olaparib treatment (Supplementary Fig. 1i, j). Of note, blocking PD-1 with a monoclonal antibody against mouse PD-1 did not improve the response to olaparib (Fig. 1c), suggesting that BP tumors are protected from T cell-mediated destruction.

Further analysis by flow cytometry revealed that BP tumors had only a small proportion of T cells, but contained a large population of tumor-associated macrophages (TAMs; CD45^+^ CD11b^+^ F4/80^+^), which was not significantly affected by olaparib treatment (Fig. 1d). Strikingly, M2-like (MHC-II^Low^ CD206^+^) macrophages were the dominant TAM sub-population in both control and olaparib-treated groups (Fig. 1d), suggesting an immunosuppressive TAM population developed in BP tumors independently of olaparib treatment. To confirm the immunosuppressive function of these TAMs, we harvested TAMs from BP breast tumors and co-cultured them with splenic CD8^+^ T cells isolated from naïve mice (Fig. 1e). Indeed, CD8^+^ T cells co-cultured with TAMs had significantly reduced IFNγ, TNFα and Granzyme B production, as well as decreased surface expression of CD25 and lower levels of effector cells (CD44^high^CD62L^low^), as compared to controls (Fig. 1f, g).

To determine whether immunosuppressive TAMs are more predominant in BRCA1-deficient breast cancers than in ovarian cancers, we compared treatment-naïve mouse breast and ovarian BRCA1-deficient tumors. As shown in Supplementary Fig. 1k, the M2/M1 ratio of TAMs was significantly higher in BP breast tumors compared to PBM ovarian tumors. To confirm this observation was not specific to mouse tumor models, we analyzed patient data from The Cancer Genome Atlas (TCGA) as well as additional datasets from NCBI’s gene expression omnibus (GEO). In line with our mouse tumor results, patient *BRCA*1-mutant breast tumors have significantly higher enrichment scores of M2 macrophage gene signature^30,31^ than *BRCA1*-mutant ovarian tumors (Supplementary Fig. 1l, m). Further, gene set enrichment analysis (GSEA) of signatures or signaling pathways associated with M2-like macrophage polarization or an immunosuppressive TIME^32,33^, such as epithelial mesenchymal transition (EMT), STAT3 targets, angiogenesis, hypoxia, TGF-beta signaling, and KRAS signaling, were upregulated in *BRCA1*-mutant breast tumors compared to *BRCA1*-mutant ovarian tumors (Supplementary Fig. 1n, o). These data indicate that the immunosuppressive nature of TAMs and the TIME is different between BRCA1-deficient breast and ovarian cancers.

### BRCA1-deficiency contributes to M2-like macrophage polarization in breast cancers

**Fig. 2:**
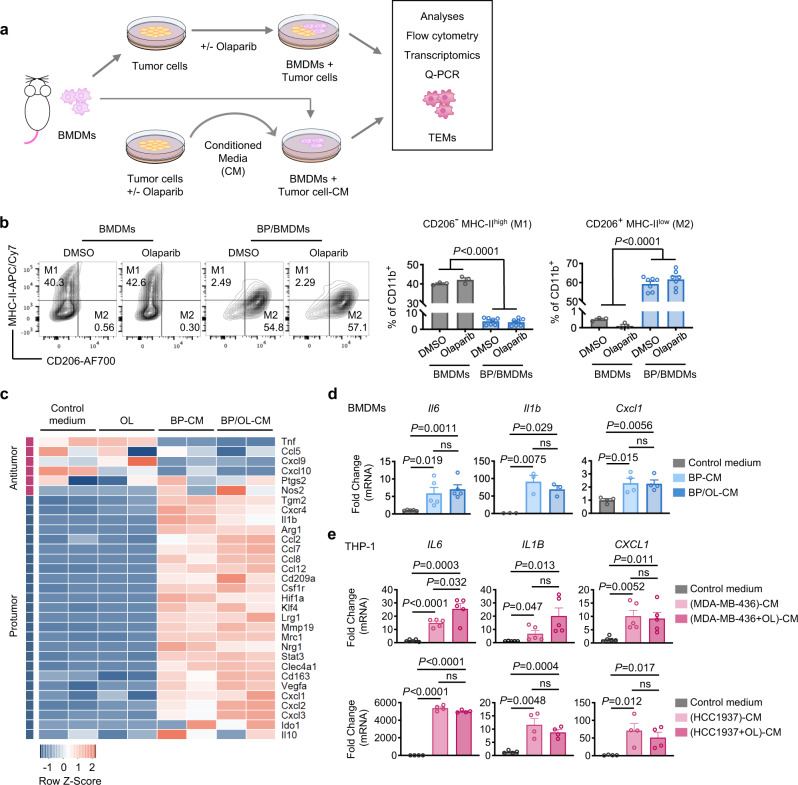
BRCA1-deficient breast tumor cells induce M2-like macrophage polarization in vitro. **a** Diagram of workflow. Top, mouse bone marrow-derived macrophages (BMDMs) co-cultured with tumor cells with or without olaparib treatment. Bottom, BMDMs were incubated with 50% conditioned media (CM) harvested from olaparib- or DMSO-treated tumor cells. TEMs, tumor cell-educated macrophages. **b** Flow cytometry analysis of BMDMs co-cultured with BP tumor cells with or without olaparib (5 μM) for 2 days. BMDMs (CD11b^+^) were plotted as CD206 versus MHC-II to identify M1-like (CD206^-^ MHC-II^high^) and M2-like (CD206^+^ MHC-II^low^) polarization phenotypes (BMDMs, *n* = 3; BP/BMDMs, *n* = 7). Flow cytometry plot axes are displayed in logarithmic scale. **c** Heat map of gene expression for anti-tumor and pro-tumor genes in BMDMs incubated with DMSO vehicle control, olaparib (OL, 5 μM), 50% BP-CM, or 50% OL-treated BP (BP/OL)-CM for 24 h (*n* = 2 for each group). **d** RT-qPCR analysis of mouse BMDMs incubated with control medium, 50% BP-CM, or 50% BP/OL-CM for 24 h (*Il6*, *n* = 5; *Il1b*, *n* = 3; *Cxcl1*, *n* = 4). **e** RT-qPCR analysis of THP-1 human macrophages incubated with control medium, 50% tumor cell-CM, or 50% CM of olaparib-treated tumor cells for 24 h (MDA-MB-436, *n* = 5; HCC1937, *n* = 4). Data are presented as mean ± SEM. One-way ANOVA (**b**). Two-tailed unpaired *t* test or Mann–Whitney test (**d**) and (**e**). ns, not significant. Source data are provided as a Source Data file.

Given the highly M2-like nature of TAMs in BRCA1-deficient breast tumors, we next examined the interaction of macrophages and tumor cells in vitro in the presence or absence of olaparib. We first established a co-culture system with mouse bone marrow-derived macrophages (BMDMs) and primary BP tumor cells with or without olaparib and termed the resulting macrophages as tumor cell-educated macrophages (TEMs) (Fig. 2a). Strikingly, we found that co-culturing with BP tumor cells induced a dramatic reduction of the M1-like population with a concurrent substantial increase in the M2-like macrophage population. Of note, olaparib exerted no significant effect on macrophage polarization after 2 days or 5 days of treatment (Fig. 2b and Supplementary Fig. 2a, b). Likewise, co-culturing *BRCA1*-mutant breast cancer MDA-MB-436 cells with human THP-1 macrophages showed that MDA-MB-436 cells significantly increased M2-like polarization of THP-1, as shown by up-regulation of CD163 (an M2 marker) and reduction of CD86 (an M1 marker), which was not significantly affected by olaparib (Supplementary Fig. 2c). Consistent with our results described above, BRCA1-deficient BP breast tumor cells more potently polarized BMDMs into M2-like macrophages compared to BRCA1-deficient PBM ovarian tumor cells (Supplementary Fig. 2d).

To investigate whether BRCA1-deficiency contributes to M2-like polarization in breast cancers, we co-cultured BP and BP + BRCA1 cells with BMDMs. Our results showed that while both BP and BP + BRCA1 cells significantly induced M2-like polarization of BMDMs, BP cells promoted M2-like polarization significantly more potently than BP + BRCA1 cells (Supplementary Fig. 2e). We also generated isogenic pairs of BRCA1-deficient and -reconstituted human breast cancer cell lines MDA-MB-436 and HCC1937. Both BRCA1-deficient and -reconstituted human breast cancer cell lines significantly increased M2-like polarization of THP1 macrophages in co-culture experiments (Supplementary Fig. 2f–i). Notably, BRCA1 restoration in both MDA-MB-436 and HCC1937 cells significantly inhibited the up-regulation of CD163 with little effect on the reduction of CD86 in THP1 macrophages, suggesting that BRCA1 restoration reduced the ability of BRCA1-deficient breast tumor cells to promote the M2-like polarization (Supplementary Fig. 2g, i). Previous studies have shown that varied oncogenic events, e.g., loss of p53 and loss of PTEN, are able to induce the formation of pro-tumor macrophages in breast cancer^34–36^. Our results suggest that loss of BRCA1 also contributes to the potential of breast cancer cells to promote pro-tumor M2-like macrophage polarization.

Tumor cell/macrophage co-culture systems involve continuous autocrine and paracrine signaling, as well as direct cell–cell interactions, between these two cell types. To deconvolute this system, we incubated BMDMs with control medium or conditioned media (CM) from BP tumor cells (BP-CM) or olaparib-treated BP cells (BP/OL-CM) (Fig. 2a). Of note, both BP-CM and BP/OL-CM promoted an M2-like polarization that resulted in a significant increase of the M2/M1 ratio, albeit less robust than that of the tumor cell/macrophage co-culture systems (Supplementary Fig. 2j).

We next conducted RNA-seq analysis of BMDMs treated with control medium, olaparib, BP-CM or BP/OL-CM. As shown in Fig. 2c, olaparib had little effect on BMDMs. By contrast, BP-CM strongly up-regulated genes associated with an M2-like/pro-tumorigenic phenotype (e.g., *Arg1*, *Ccl2*, *Ccl8*, *Mrc1*, and *Vegfa*), while it down-regulated expression of some M1-like/anti-tumorigenic genes (e.g., *Tnf* and *Cxcl10*). Of note, BMDMs had a similar transcriptional profile when they were treated with BP/OL-CM to that seen with BP-CM (Fig. 2c). We further confirmed by RT-qPCR that the expression of several critical pro-tumorigenic genes, including *Il6, Il1b* and *Cxcl1*, were significantly increased in BMDMs treated with BP-CM or BP/OL-CM relative to control medium (Fig. 2d). To assess whether this could be recapitulated by human *BRCA1*-mutant breast cancer cells, we treated THP-1 macrophages with CM collected from *BRCA1*-mutant breast cancer cell lines MDA-MB-436 or HCC1937. Consistently, THP-1 macrophages treated with CM of tumor cells or CM of olaparib-treated tumor cells showed significantly up-regulated *IL6*, *IL1B* and *CXCL1* gene expression (Fig. 2e). These data suggest that *BRCA1*-deficient breast tumor cells can “educate” macrophages to become M2-like pro-tumorigenic macrophages independent of PARPi. To assess the immunosuppressive function of TEMs, we generated TEMs by incubating BMDMs with BP-CM for 2 days, followed by co-culturing with splenic CD8^+^ T cells isolated from naïve mice. Our results showed that TEMs, but not control BMDMs, significantly reduced IFNγ, TNFα and Granzyme B production by CD8^+^ T cells, suggesting that BP TEMs acquire an immunosuppressive function (Supplementary Fig. 2k).

### TEMs/TAMs suppress olaparib-induced DNA damage in *BRCA1*-deficient breast tumor cells

**Fig. 3:**
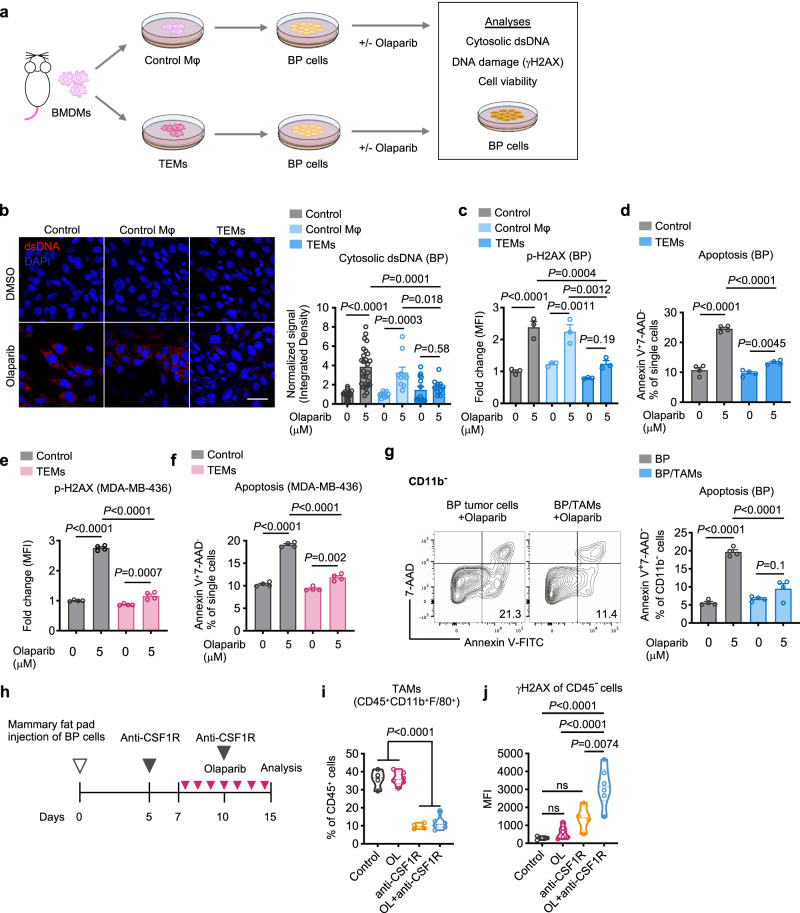
TEMs/TAMs suppress olaparib-induced DNA damage in BRCA1-deficient breast tumor cells. **a** Diagram of workflow for (**b**)–(**f**). Conditioned media from control macrophages (Mφ) or tumor-educated macrophages (TEMs) were added to tumor cell cultures, followed by olaparib treatment. **b** BP cells were stained using DAPI and an anti-dsDNA antibody after two days of olaparib treatment. The intensity of dsDNA fragments in the cytosol was quantified. Scale bar, 50 μm (control, *n* = 21 fields; olaparib, *n* = 26 fields; control macrophages, *n* = 10 fields; control macrophages + olaparib, *n* = 10 fields; TEMs, *n* = 14 fields; TEMs + olaparib, *n* = 10 fields examined over two independent experiments). **c** BP cells were stained with anti-H2AX phospho-Ser139 antibody and analyzed by flow cytometry. MFI, median florescence intensity (*n* = 3). **d** BP cells were analyzed for apoptosis (Annexin V^+^ 7-AAD^−^) (*n* = 4). **e** MDA-MB-436 cells were stained with anti-H2AX phospho-Ser139 antibody and analyzed by flow cytometry (*n* = 4). **f** MDA-MB-436 cells were analyzed for apoptosis (Annexin V^+^ 7-AAD^−^) (*n* = 4). **g** Apoptotic analyses of BP cells co-cultured with or without TAMs sorted from BP tumors, followed by three days of olaparib treatment (*n* = 4). Flow cytometry plot axes are displayed in logarithmic scale. **h** Schematic representation of the experiments for (**i**) and (**j**). **i** and **j** Analysis of TAMs (**i**) and γ-H2AX in CD45-negative cells (**j**) in BP tumors after two IP injections of anti-CSF1R antibody and 7 days of treatment with olaparib (OL) as monotherapy or in combination. Control, *n* = 4; OL, *n* = 6; anti-CSF1R, *n* = 4; OL + anti-CSF1R, *n* = 6. Each dot represents data from a single tumor. Data are presented as mean ± SEM (**b**)–(**g**) or median with quartiles (violin plots, **i** and **j**). One-way ANOVA. ns, not significant. Source data are provided as a Source Data file.

We next sought to investigate how M2-like TEMs affect tumor cells’ response to PARPi (Fig. 3a). Since the synthetic lethal response upon PARPi treatment is driven by highly deleterious DNA double-strand breaks (DSBs)^1^, we first tested how TEMs affect PARPi-induced DSBs in tumor cells. BP tumor cells were incubated with CM from TEMs or naïve BMDMs (control Mφ), followed by olaparib treatment, and analyzed by immunofluorescence (IF) with an antibody against double-stranded DNA (dsDNA). As expected, olaparib induced accumulation of dsDNA fragments in the cytosol of BP cells, while CM from TEMs, but not naïve BMDMs, suppressed the production of dsDNA fragments in BP cells upon olaparib treatment (Fig. 3b). We then validated our findings using human TEMs generated via co-culturing of THP-1 macrophages with MDA-MB-436 cells. Consistently, CM derived from the TEMs significantly reduced the amount of cytosolic dsDNA fragments in MDA-MB-436 cells induced by olaparib treatment (Supplementary Fig. 3a). We further assessed DNA damage by measuring histone H2AX phosphorylation at Serine 139 (γ-H2AX), a surrogate marker and an early cellular response to DNA DSBs^37^. Notably, BP tumor cells responded to olaparib with an increase of γ-H2AX, which was significantly reduced by CM from TEMs, but not control BMDMs (Fig. 3c and Supplementary Fig. 3b, c). In line with these findings, CM from TEMs also decreased olaparib-induced apoptosis of mouse BP tumor cells by approximately 40–50% (Fig. 3d and Supplementary Fig. 3d). Similarly, MDA-MB-436-educated THP-1 macrophages significantly inhibited olaparib-induced γ-H2AX up-regulation as well as apoptosis in MDA-MB-436 cells (Fig. 3e, f). Given the striking finding that CM derived from TEMs were able to significantly reduce olaparib-induced DNA damage and cell death, we sought to identify potential soluble factors released from TEMs that could mediate these effects. We derived sufficient mouse TEMs or human TEMs via co-culturing of mouse BMDMs with BP cells, or THP-1 macrophages with MDA-MB-436 cells. The CM collected from these TEMs were fractionated using a centrifugal filter device with a 3 kDa molecular weight cut-off membrane. Surprisingly, we found that soluble factors of <3 kDa in TEM-CM are responsible for protecting against DNA damage and cell death induced by olaparib in both mouse BP cells and human MDA-MB-436 cells (Supplementary Fig. 3e–h).

We also harvested TAMs directly from BP tumors to assess their effects on tumor cells ex vivo. Indeed, TAMs significantly inhibited BP cell death and reduced accumulation of dsDNA in the cytoplasm of BP cells following treatment with olaparib (Fig. 3g and Supplementary Fig. 3i). To confirm these findings in vivo, we depleted TAMs by treating BP tumor-bearing mice with an anti-CSF1R antibody (Fig. 3h, i). Our results showed that depletion of TAMs significantly increased the amount of cytosolic dsDNA and γ-H2AX in tumor cells upon olaparib treatment (Supplementary Fig. 3j and Fig. 3j). These data suggest that pro-tumorigenic TEMs/TAMs suppress the DNA damage and synthetic lethal response of BRCA1-deficient breast tumor cells to olaparib.

### STING activation reprograms pro-tumor macrophages and diminishes TEMs-mediated protection of tumor cells from olaparib

**Fig. 4:**
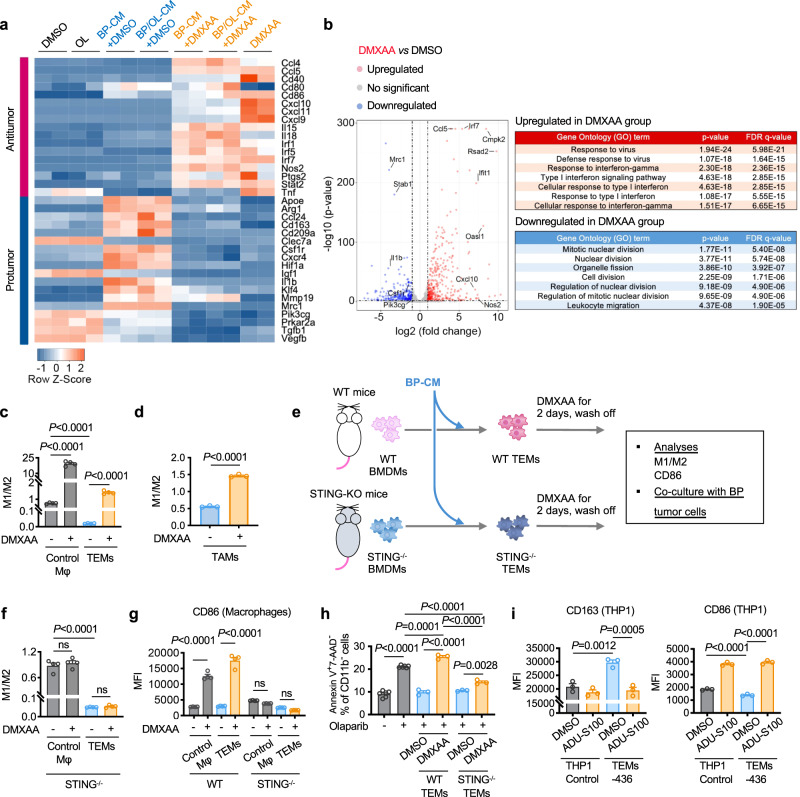
STING agonists can reprogram M2-like TEMs/TAMs into an M1-like state. **a** and **b** Mouse BMDMs were subjected to transcriptome analysis after treatment for 24 h with DMSO vehicle control, olaparib (OL, 5 μM), DMXAA (0.05 mg/mL), 50% BP-CM with or without DMXAA, or 50% BP/OL-CM with or without DMXAA. **a** Heat map of anti-tumor and pro-tumorigenic gene expression in BMDMs (n = 2 for each group). **b** Left, volcano plot showing the significance and magnitude of changes in gene expression of BMDMs treated with BP-CM/DMXAA compared to BP-CM/DMSO (*n* = 2 for each group). Statistical significance was calculated using a two-sided Wald test and adjusted for multiple testing using the Benjamini–Hochberg procedure. Right, top-ranked up-regulated and down-regulated gene ontology (GO) terms in BMDMs treated with BP-CM/DMXAA. Significance of enriched terms were adjusted using the Benjamini–Hochberg procedure for multi-testing. **c** Analysis of control macrophages (Mφ) and BP TEMs treated with or without DMXAA (0.05 mg/mL) for 2 days. M1-like (CD11b^+^ CD206^-^ MHC-II^high^) to M2-like (CD11b^+^ CD206^+^ MHC-II^low^) ratio was analyzed by flow cytometry (*n* = 4). **d** Analysis of M1 to M2 ratio of TAMs (sorted from BP tumors) treated with or without DMXAA (0.05 mg/mL) for 2 days (*n* = 3). **e** Diagram of workflow for (**f-h**). BMDMs isolated from wildtype (WT) or STING knockout (STING^-/*-*^) C57/BL6J mice were incubated with control medium or 50% BP-CM to generate control naïve BMDMs and TEMs, respectively. Cells were then treated with or without DMXAA (0.05 mg/mL) for 2 days. **f** and **g** M1 to M2 ratio (**f**) and surface levels of the co-stimulatory molecule CD86 (**g**) were analyzed by flow cytometry (*n* = 4). **h** BP cells were co-cultured with or without WT TEMs or STING^−/−^ TEMs pre-treated with or without DMXAA (0.05 mg/mL), followed by 2 days of olaparib (5 μM) treatment. BP cells were then analyzed for apoptosis (Annexin V^+^ 7-AAD^−^; BP cells, *n* = 6; BP + WT TEMs, *n* = 3; BP + STING^−/−^ TEMs, *n* = 3). **i** Expression of M2 (CD163) and M1 (CD86) markers in control THP-1 macrophages or MDA-MB-436 tumor cell-educated THP-1 macrophages (TEMs-436) treated with or without ADU-S100 (10 μM) for two days (*n* = 3). Data are presented as mean ± SEM. One-way ANOVA (**c**) and (**f**)–(**i**). Two-tailed unpaired *t* test (**d**). ns, not significant. Source data are provided as a Source Data file.

We have previously shown that activation of the STING pathway in myeloid cells triggered by dsDNA fragments released from BRCA1-deficient ovarian tumor cells upon PARPi is required for the anti-tumor activity of PARPi^16^. Since BRCA1-deficient breast tumor cells have reduced production of dsDNA fragments upon PARPi in the presence of TEMs/TAMs, we wondered whether an exogenous STING agonist could alter macrophage state and tumor cell response to PARPi. We first investigated whether DMXAA, a potent murine STING agonist^38,39^, could inhibit pro-tumorigenic polarization of macrophages by BP tumor cells. We performed further transcriptomic analyses of BMDMs treated with control, olaparib, BP-CM or BP/OL-CM in the presence or absence of DMXAA (Fig. 4a). Notably, DMXAA not only suppressed tumor cell-induced up-regulation of genes associated with pro-tumorigenic M2 polarization, including *Arg1*, *Csf1r*, *Il1b* and *Mrc1*, but also strongly stimulated the expression of genes associated with an anti-tumorigenic M1 signature (e.g., *Ccl5*, *Cxcl10*, *Cd40*, *Cd86, Il18* and *Nos2*) (Fig. 4a). Gene ontology (GO) analysis revealed that DMXAA significantly increased “response to virus”, “response to interferon-gamma” and “type I interferon signaling pathway” signatures, and concurrently decreased “mitotic nuclear division” and “organelle fission” biological processes in these macrophages (Fig. 4b and Supplementary Fig. 4a). In keeping with changes in gene expression, macrophage activation was also reflected in morphological changes. As shown in Supplementary Fig. 4b, BMDMs incubated with BP-CM showed an elongated or stellate morphology, which was similar to the M2-like macrophages induced by IL4^40^. In contrast, addition of DMXAA changed these cells to a “fried egg” shape (round-shaped cells with large nuclei centered in the cytoplasm), which was similar to the morphology of M1-like macrophages induced by combined LPS and IFNγ^40^. These data indicate that pharmacological activation of the STING pathway can shift polarization of BMDMs toward an M1-like state even in the presence of BP-CM.

Given our finding that STING activation could promote M1-like macrophage polarization, we then asked whether a STING agonist could reverse M2-like TEMs to M1-like macrophages. To this end, we first derived TEMs by incubating BMDMs with BP-CM, and then treated these TEMs with a STING agonist. Indeed, DMXAA not only promoted M1-like polarization of control BMDMs but also reversed M2-like TEMs to M1-like macrophages with significantly increased STING pathway activation (Fig. 4c and Supplementary Fig. 4c), consistent with previous studies^41,42^. Notably, DMXAA restored DNA damage response in BP cells upon olaparib treatment in the presence of TEMs (Supplementary Fig. 4d, e). Furthermore, DMXAA was also able to reprogram M2-like TAMs harvested from BP tumors ex vivo (Fig. 4d).

To further investigate the role of the STING pathway in macrophage polarization and reprogramming, we isolated BMDMs from STING-knockout (STING^−/−^) goldenticket (gt) mice (*Tmem173*^gt/gt^) and co-cultured with BP cells ex vivo (Fig. 4e). Although STING^−/−^ BMDMs were readily polarized to M2-like TEMs when treated with BP-CM, DMXAA did not induce M1-like polarization of STING^−/−^ control BMDMs or STING^−/−^ TEMs (Fig. 4f), suggesting that STING in macrophages is not required for M2-like polarization, but is necessary for M1-like reprogramming. Consistently, DMXAA significantly increased the expression of the co-stimulatory molecule CD86 in WT control BMDMs and WT TEMs, but not in STING^−/−^ control BMDMs or STING^−/−^ TEMs (Fig. 4g). In addition, we evaluated the effect of DMXAA on TEM-mediated protection of tumor cells from olaparib. As shown in Fig. 4h, pre-treatment of WT TEMs with DMXAA abrogated TEM-mediated protection of tumor cells against olaparib-induced apoptosis. By contrast, pre-treatment of STING^−/−^ TEMs with DMXAA only modestly reduced STING^−/−^ TEMs-mediated protection from olaparib (Fig. 4h). This result suggests that, while DMXAA reprograms TEMs largely through the STING pathway, DMXAA may also affect macrophage activity in a STING-independent manner, which modestly attenuated TEM-mediated protection from olaparib. We next tested whether a STING agonist could also reprogram human TEMs. We derived THP-1 TEMs by co-culturing THP-1 macrophages with MDA-MB-436 tumor cells, and then treated THP-1 TEMs with ADU-S100, a human STING agonist. Indeed, ADU-S100 promoted a shift from M2-like to M1-like phenotype, as shown by suppressed CD163 up-regulation and increased expression of CD86 on THP-1 TEMs (Fig. 4i).

### STING agonists improve therapeutic response of orthotopic BP tumors to olaparib in syngeneic immunocompetent mice in vivo

**Fig. 5:**
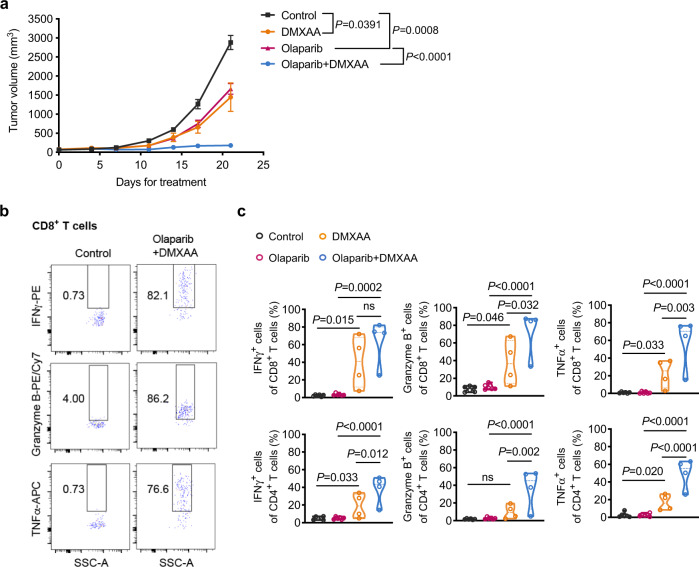
STING agonists improve therapeutic response of orthotopic BP tumors to olaparib in syngeneic immunocompetent mice in vivo. **a** BP tumor growth in FVB mice treated with olaparib (50 mg/kg, IP, QD) or intratumoral (IT) injections of DMXAA (10 mg/kg, one dose per week for 3 weeks [total of 3 doses]) as monotherapy or in combination. Control, *n* = 8; olaparib, *n* = 9; DMXAA, *n* = 6; olaparib + DMXAA, *n* = 7. **b** and **c** Analysis of BP tumors after 21 days of treatment for effector cytokine production by intratumoral CD8^+^ T cells and CD4^+^ T cells. Flow cytometry plot axes are displayed in logarithmic scale (**b**). Control, *n* = 6; olaparib, *n* = 6; DMXAA, *n* = 4; olaparib + DMXAA, *n* = 4. Each dot represents data from a single tumor (**c**). Data are presented as mean ± SEM (**a**), or median with quartiles (violin plots, **c**). Two-way ANOVA (**a**). One-way ANOVA (**c**). ns, not significant. Source data are provided as a Source Data file.

To determine whether stimulation of the STING pathway could potentiate the anti-tumor activity of PARPi in vivo, we established a cohort of FVB female mice bearing orthotopic BP tumors. Tumor-bearing mice were randomized into four groups and subjected to control, olaparib, DMXAA or to a combination treatment with olaparib and DMXAA. DMXAA was administered to BP tumor-bearing mice via intratumoral (IT) injection, as this delivery method has shown promising potential^39^. A relatively low dose of DMXAA (10 mg/kg) was administered once per week for 3 weeks (total of 3 doses). While DMXAA and olaparib monotherapy induced modest tumor growth inhibition, combined treatment strongly suppressed tumor growth (Fig. 5a). Analysis of the tumor immune infiltrate showed that DMXAA monotherapy up-regulated production of anti-tumor cytokines (i.e., IFNγ, Granzyme B, and TNFα) in both CD8^+^ and CD4^+^ T cells, which was further enhanced by combining with olaparib therapy (Fig. 5b, c). Moreover, depletion of TAMs or CD8^+^ T cells in the presence of DMXAA significantly reduced the anti-tumor activity of the combination therapy, confirming the contributions of these immune cells to the therapeutic efficacy (Supplementary Fig. 5a). These results suggest that STING agonists overcome immune suppression and markedly improve the response of *Brca1*-deficient breast tumors to olaparib in vivo.

Mehta et al. recently showed that PARP inhibition induces CSF1R-dependent immune-suppressive macrophages^43^. Hence, we also investigated the efficacy of PARPi in combination with a monoclonal antibody against mouse CSF1R in orthotopic allografts of BP tumors. Indeed, combined treatment with olaparib and anti-CSF1R resulted in improved efficacy compared to anti-CSF1R or olaparib alone (Supplementary Fig. 5b). However, this improved therapeutic efficacy of combined olaparib and anti-CSF1R was less pronounced than that of combined olaparib and DMXAA (Fig. 5a and Supplementary Fig. 5b). In agreement, the enhancement of intratumoral CD8^+^ and CD4^+^ T cell activation was less significant in tumors treated with combined olaparib and anti-CSF1R compared to tumors treated with combined olaparib and DMXAA (Fig. 5b, c and Supplementary Fig. 5c, d). Further analysis revealed that, while anti-CSF1R antibody depleted TAMs (Supplementary Fig. 5e), DMXAA did not alter the abundance of TAMs; instead, it strongly shifted TAMs from M2-like macrophages into an M1-like state (Supplementary Fig. 5f, g). These data indicate that STING agonist-mediated TAM reprogramming provides a potentially superior approach to harness TAMs for the treatment of immunosuppressive cancers.

### Systemic delivery of STING agonists potentiates the therapeutic efficacy of olaparib independently of tumor cell-intrinsic STING

**Fig. 6:**
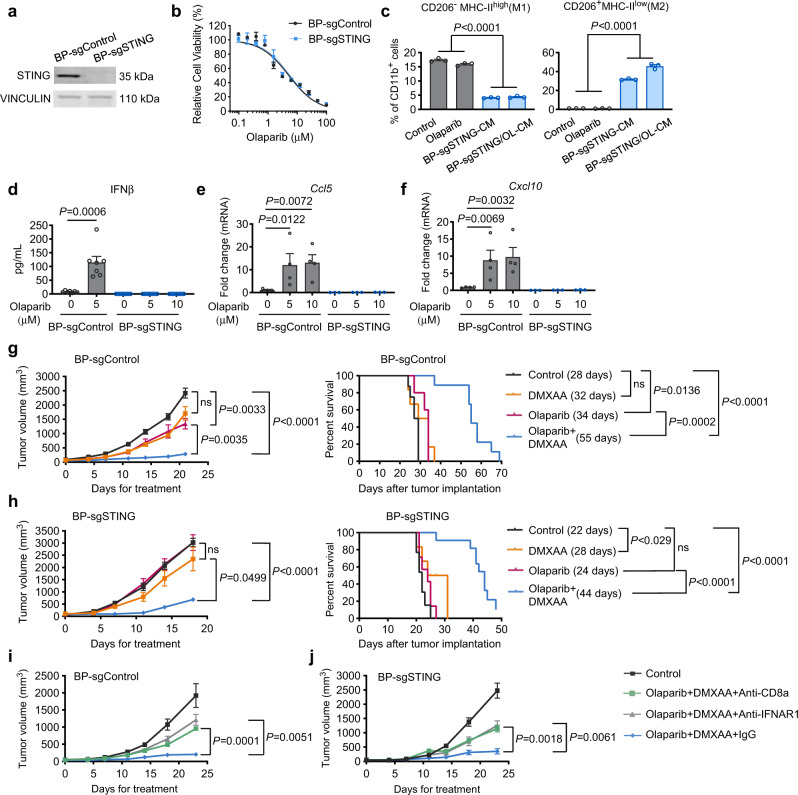
Systemic delivery of a STING agonist sensitizes STING-null BP tumors to olaparib in vivo. **a** Western blots for STING and VINCULIN in CRISPR/Cas9 control and STING knockout BP tumor cells (BP-sgControl and BP-sgSTING). Representative blots of two independent experiments are shown. **b** CellTiter-Glo analysis showing cell viability of BP-sgControl and BP-sgSTING cells after 3 days of treatment with serial dilution of olaparib (*n* = 3). **c** Flow cytometry analysis of mouse BMDMs treated with DMSO vehicle control, olaparib (OL, 5 μM), 50% BP-sgSTING-CM, or 50% BP-sgSTING/OL-CM for two days (*n* = 3). **d** ELISA analysis of IFNβ in media from BP-sgControl or BP-sgSTING cells with or without 2 days of olaparib treatment (*n* = 7). **e** and **f** RT-qPCR analysis of *Ccl5* (**e**) and *Cxcl10* (**f**) in BP-sgControl and BP-sgSTING cells treated with or without olaparib for 2 days (BP-sgControl, *n* = 4; BP-sgSTING, *n* = 3). **g** Tumor growth (left) and survival (right) of BP-sgControl tumor-bearing FVB mice treated with olaparib (50 mg/kg, IP, QD), DMXAA (10 mg/kg, IP) or olaparib + DMXAA. Median survivals are shown in parentheses. Left, Control, *n* = 13; Olaparib, *n* = 7; DMXAA, *n* = 9; Olaparib + DMXAA, *n* = 14. Right, Control, *n* = 8; olaparib, *n* = 5; DMXAA, *n* = 6; olaparib + DMXAA, *n* = 9. **h** Tumor growth (left) and survival (right) of BP-sgSTING tumor-bearing FVB mice treated with olaparib (50 mg/kg, IP, QD), DMXAA (10 mg/kg, IP) or olaparib + DMXAA. Median survivals are shown in parentheses. Left, Control, *n* = 24; olaparib, *n* = 11; DMXAA, *n* = 9; olaparib + DMXAA, *n* = 19. Right, Control, *n* = 13; olaparib, *n* = 7; DMXAA, *n* = 6; olaparib + DMXAA, *n* = 11. **i** and **j** BP-sgControl (**i**) and BP-sgSTING (**j**) tumor growth in FVB mice treated with olaparib + DMXAA with or without anti-CD8 or anti-IFNAR1 neutralizing antibodies (*n* = 6 per condition). Data are presented as mean ± SEM. One-way ANOVA (**c, e**, and **f**). Two-tailed Mann–Whitney test (**d**). Two-way ANOVA for tumor growth (**g**)–(**j**). Log-rank Mantel–Cox test for survival (**g** and **h**). ns, not significant. Source data are provided as a Source Data file.

We next sought to investigate whether STING in tumor cells is required for the therapeutic response to combined olaparib and a STING agonist. We generated STING-null BP cells via CRISPR/Cas9-mediated gene editing, and the resulting cells were termed BP-sgSTING or BP-sgControl (Fig. 6a). Loss of tumor cell-intrinsic STING did not affect the synthetic lethal response to olaparib in vitro (Fig. 6b). BP-sgSTING cells (either with or without olaparib treatment) were still able to strongly promote M2-like polarization of macrophages (Fig. 6c). However, deletion of STING in BP cells abrogated olaparib-induced up-regulation of pro-inflammatory cytokines, including *Ifnb*, *Ccl5* and *Cxcl10*, which have been associated with immune activation and CD8^+^ T cell recruitment (Fig. 6d–f), consistent with previous reports^17,44^. Notably, unlike STING-proficient BP tumors that responded to DMXAA and its combination with olaparib (Fig. 5), BP-sgSTING tumors were refractory to locally administered DMXAA via IT injection as a single agent or in combination with olaparib (Supplementary Fig. 6a), consistent with recently reported findings^44^. To explore this further, we compared the TIME of STING-deficient and STING-proficient BP tumors without treatment. Our results revealed that BP-sgSTING tumors have significantly reduced overall abundance of CD45^+^ leukocytes, including TAMs and DCs (Supplementary Fig. 6b). This is consistent with the finding that BP-sgSTING cells have much reduced intrinsic immunity than STING-proficient BP cells (Fig. 6d–f), which may underlie their reduced capacity to recruit immune cells into the tumor microenvironment. Therefore, the number of available immune cells within BP-sgSTING tumors is likely too small for a locally delivered STING agonist to act effectively. Indeed, olaparib plus IT injection of DMXAA did not induce activation of CD8^+^ T cells in BP-sgSTING tumors (Supplementary Fig. 6c).

Given recent findings demonstrating that a systemically administered STING agonist leads to DC and T cell activation in spleens and tumor-draining lymph nodes (TDLNs), which may contribute to anti-tumor immunity^45^, we next asked whether systemic administration of a STING agonist could potentiate anti-tumor immunity and synergize with PARPi. Hence, for these studies DMXAA (10 mg/kg) was administered weekly via intraperitoneal (IP) injection. Whereas IP injection of DMXAA as a single agent had no significant impact on the growth of BP-sgControl or BP-sgSTING tumors, the combination of systemic DMXAA administration with olaparib resulted in strong inhibition of tumor growth, and significantly prolonged survival of both BP-sgControl and BP-sgSTING tumor-bearing mice (Fig. 6g, h). Consistently, analysis of intratumoral immune cells from BP-sgSTING tumors revealed that, compared to the control group, CD8^+^ T cell infiltration, anti-tumor cytokine production by CD8^+^ T cells, and the proportion of effector CD8^+^ T cells were significantly increased by combined treatment with olaparib and IP injection of DMXAA (Supplementary Fig. 6c), suggesting that a systemically delivered STING agonist was able to activate and mobilize host immune cells to exert anti-tumor immunity. We then carried out an additional experiment by treating tumor-bearing mice with IFNAR1 or CD8 blocking antibodies. As shown in Fig. 6i, j, the efficacy of the combination therapy was significantly mitigated, but not completely prevented, by IFNAR1 or CD8 neutralization. These results suggest that both innate and adaptive immune function contribute to the anti-tumor activity driven by olaparib in combination with a STING agonist. Together, our data provide compelling evidence that combination of olaparib with systemic delivery of a STING agonist overcomes the resistance of STING-null BRCA1-deficient breast tumors to PARPi.

### Host STING is essential for the therapeutic efficacy of combined olaparib and STING agonist

To investigate the contribution of host STING to the anti-tumor efficacy of PARPi plus a STING agonist in *Brca1*-deficient breast tumors, we employed STING^−/−^ mice (*Tmem173*^gt/gt^, C57BL/6J) and EO771 murine breast cancer cells, a syngeneic mouse model on C57BL/6 background. As BRCA1 was readily detected in EO771 cells, we proceeded to generate *Brca1*-deficient EO771 cells using CRISPR/Cas9 (Supplementary Fig. 6d). As expected, EO771-sgBRCA1 cells exhibited significantly increased sensitivity to olaparib compared to EO771-sgControl cells (Supplementary Fig. 6e). We then compared the response of orthotopic *Brca1*-deficient EO771 tumors to the combination therapy in WT and STING^−/−^ C57BL/6J mice. Our results showed that olaparib combined with systemic delivery of DMXAA via IP injection strongly inhibited tumor growth in WT mice (Supplementary Fig. 6f). By contrast, the combination therapy showed little anti-tumor growth effect in STING-KO mice (Supplementary Fig. 6f), suggestive of an essential role of host STING in the therapeutic efficacy. In parallel, we generated EO771-sgBRCA1-sgSTING cells using CRISPR/Cas9 to evaluate response to combined olaparib and DMXAA in WT C57BL/6J mice (Supplementary Fig. 6g). In this case, EO771 tumors with concurrent deletion of BRCA1 and STING responded strongly to combined olaparib and systemic DMXAA administered via IP injection in WT mice (Supplementary Fig. 6h), thus showing that tumor cell-intrinsic STING is not critical for the therapeutic efficacy of this combination therapy. We also found that the combination therapy with IP injection of DMXAA showed markedly improved therapeutic efficacy against EO771-sgBRCA1-sgSTING tumors over the combination therapy with DMXAA delivered via IT injection (Supplementary Fig. 6i).

## Discussion

**Fig. 7:**
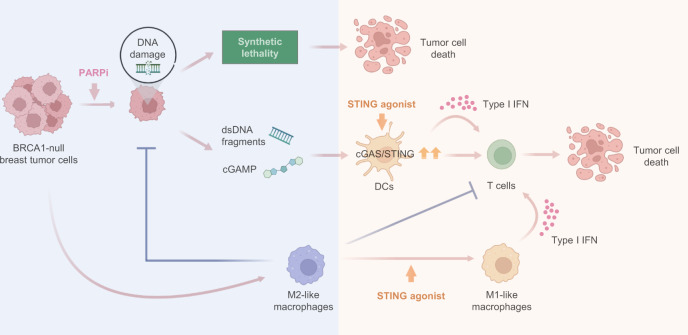
Harnessing anti-tumor immunity with STING agonists overcomes immune suppression and resistance to PARP inhibition in BRCA1-deficient breast cancer. BRCA1-deficient breast tumors elicit pro-tumorigenic macrophage polarization. In turn, these tumor-educated macrophages not only exhibit suppressive activity against T cells, but also attenuate PARPi-mediated synthetic lethality and the production of double-stranded DNA (dsDNA) fragments, thus diminishing the activation of the DNA sensing adaptor STING and rendering BRCA1-deficient breast tumors resistant to PARPi therapy (blue shading). Exogenous agonists of the STING pathway reprogram the macrophages and trigger innate immune activation of both macrophages and DCs, potentiating PARPi therapy to induce tumor cell DNA damage and an adaptative immune response that re-sensitizes tumors to PARPi therapy (orange shading).

PARPi have markedly improved overall survival of patients with *BRCA*-mutated advanced ovarian cancer^6^. While the OlympiA trial recently reported a significantly longer invasive disease-free and overall survival of adjuvant olaparib in *BRCA*-mutated early-stage breast cancer^9^, neither olaparib nor talazoparib conferred overall survival benefit in *BRCA*-mutated advanced breast cancer^10,11^. Here, we demonstrate that *BRCA1*-deficient breast tumor cells strongly promote M2-like polarization of TAMs independent of PARPi treatment. These TAMs not only suppress CD8^+^ T cell activation, but also significantly reduce synthetic lethal responses in PARPi-treated tumor cells and the production of dsDNA fragments, which are required for dsDNA-mediated, STING-dependent activation of antitumor immunity in the context of PARPi therapy. In agreement, Mehta et al. recently also found that TAMs rendered poor response to PARPi in *BRCA1*-deficient breast cancer^43^. The current understanding of PARPi resistance is mainly focused on tumor cell-intrinsic mechanisms, including the cellular availability of PARPi, reversion mutations in *BRCA1/2*, restoration of HR or PARylation, and DNA replication fork protection^15,46,47^. Our findings thus provide insights into the role of macrophages in PARPi therapy and demonstrate a mechanism underlying PARPi resistance that is mediated through bi-directional interactions between tumor cells and immune cells in *BRCA1*-deficient breast cancers (Fig. 7).

There is growing evidence showing TAM-mediated resistance to anti-cancer drugs^48^. For example, recent studies have reported that TAMs promote chemoresistance by suppressing Taxol-induced mitotic arrest^49^ or by inhibiting gemcitabine via release of pyrimidines that outcompete drug uptake and metabolism^50^. Despite these findings, therapeutic targeting of TAMs is challenging. CSF1R blockade is an effective approach to deplete TAMs, which depend on CSF1/CSF1R signaling for survival^51^. However, efforts to reprogram an immunosuppressive TIME with anti-CSF1R therapy have shown limited clinical benefit in advanced solid tumors due to various reasons^52–56^. In contrast to the TAM depletion strategy, reprogramming TAMs into an anti-tumor state may be a superior approach to harness the immune system against cancer. Studies have shown that dying tumor cells containing STING agonists, such as dsDNA fragments, may trigger anti-tumor immunity by activating STING signaling in macrophages^57,58^. However, this pathway may be silenced by the rapid degradation of tumor-derived DNA during TAM-mediated phagocytic clearance of apoptotic tumor cells^58,59^. In this study, we showed that a small molecule STING agonist efficiently reprogramed TAMs from a pro-tumorigenic to an anti-tumorigenic state characterized by induction of type I IFN responses and expression of the co-stimulatory molecule CD86, which may stimulate T cell cross-priming and trigger a robust adaptive anti-tumor immunity^39^. These findings are consistent with previous reports showing that macrophage depletion impairs the anti-tumor efficacy of cGAMP administration^41^, and that cyclic dinucleotides (i.e. cGAMP or c-di-AMP) down-regulate M2 markers and up-regulate M1 markers in M2-polarized BMDMs^42^. Here, we further showed that a STING agonist not only promoted anti-tumor M1-like macrophage polarization, but also inhibited pro-tumor macrophage-mediated suppression of synthetic lethality induced by PARPi, thus rendering tumors more susceptible to PARPi therapy. Indeed, PARPi in combination with STING agonists demonstrated superior anti-tumor efficacy than PARPi combined with macrophage depletion via anti-CSF1R in our syngeneic GEMM of *Brca1*-deficient breast cancers.

In addition to PARPi, other DNA damage response (DDR)-targeted agents, such as inhibitors of ATM, ATR, CHK1/2 or WEE1, are also able to produce cytoplasmic dsDNA fragments, and thus have the potential to stimulate the immune system via activation of the cGAS/STING pathway^60^. Moreover, although predominantly targeted to *BRCA*-mutant cancers, PARPi treatment for non-*BRCA*-mutant cancers is also emerging in the clinic^1^. Indeed, talazoparib has been shown to induce considerable in vitro cytotoxicity regardless of *BRCA1* mutation status, although it is still more potent in *BRCA1* mutant tumor cells^61^. Moreover, a preclinical study reported that talazoparib-induced DNA damage and STING activation independent of *BRCA* mutations in murine models of ovarian and colon cancers^62^. Further studies are needed to understand the role of TAMs in the context of different DDR targeted agents. It will also be meaningful to investigate whether and when an exogenous STING agonist should be combined with DDR-targeted therapy to induce a greater degree of STING-dependent anti-tumor activity.

Given the promising results of many pre-clinical studies, to date the majority of efforts to exploit the immunomodulatory properties of PARPi have focused on the combination of PARPi with immune checkpoint blockade (ICB), which has led to more than 25 clinical trials across different types of HR-deficient cancers, including advanced breast cancer^63^. Unexpectedly, however, initial results suggest that the combination may not effectively enhance objective response rate (ORR) compared to historical cohorts that received PARPi as a single-agent treatment^63^. Thus, it is important to better understand the mechanisms that may negate the efficacy of PARPi. Our study indicates that TAMs play an important role in influencing the response of BRCA1-deficient breast tumors to PARPi, supporting the need to assess the TIME in clinical trials. Indeed, accumulation of TAMs has been frequently observed in the T cell-excluded or inactivated TIME of advanced breast cancers^64,65^. In this study, we found that systemic delivery of a STING agonist promoted the activation of anti-tumor T cells when combined with PARPi. We further showed that host STING but not tumor cell-intrinsic STING is essential for the anti-tumor activity of the combination therapy with a systemically delivered STING agonist. These findings have potentially important clinical implications, as many solid tumors become STING-deficient during tumor progression^66–68^. The next generation of STING agonists that can be delivered systemically is currently under clinical development^45,69,70^. Our finding that systemic delivery of a STING agonist overcomes therapeutic resistance to PARP inhibition has the potential to inform the design of future clinical therapies.

## Methods

### Mice

All animal experiments described in this study were performed according to animal protocols (03-111 and 09-069) approved by the DFCI Institutional Animal Care and Use Committee (IACUC). STING knockout mice were purchased from The Jackson Laboratory (C57BL/6J-*Tmem173*^*gt*^/J, # 017537). *Brca1*^loxP/loxP^ mouse line was kindly provided by Dr. Jos Jonkers’s laboratory at Netherlands Cancer Institute. *Trp53*^loxP/loxP^ mouse line was obtained from National Cancer Institute Mouse Repository (FVB.129P2-*Trp53*^*tm1Brn*^/Nci, # 01XC2). *Brca1*^loxP/loxP^ and *Trp53*^loxP/loxP^ mouse lines were both backcrossed into the FVB/N background for more than 10 generations^16^. To develop syngeneic genetically engineered mouse models (GEMMs) of *Brca1*-deficient breast cancer, *Brca1*^loxP/loxP^ mice were further crossed with *Trp53*^loxP/loxP^ mice. The resulting 12-week-old *Brca1*^loxP/loxP^; *Trp53*^loxP/loxP^ female mice were injected intraductally with adenovirus expressing Cre recombinase under a CMV promoter, which led to the development of mammary tumors driven by concurrent loss of *Brca1* and *Trp53* (referred as BP).

### Cell culture

Cells were cultured in a humidified incubator under 5% CO_2_ at 37 °C. EO771 cell line (# 940001) was purchased from CH3 BioSystems in 2016. MDA-MB-436 (# HTB-130), HCC1937 (# CRL-2336), and THP-1 (# TIB-202) cell lines were obtained from ATCC between the years 2019 and 2020. These cell lines were tested negative for mycoplasma and authenticated using short tandem repeat analysis (Promega GenePrint 10 System). 100 μg/mL penicillin–streptomycin (Gibco) was added to all the cell cultures. EO771 was cultured in RPMI 1640 (Gibco) with 10% heat-inactivated fetal bovine serum (FBS, Gemini) and 10 mM HEPES (Gibco). MDA-MB-436 and HCC1937 cells were cultured in RPMI 1640 medium (Gibco) with 10% FBS. THP-1 monocytes were cultured in RPMI 1640 with 10% FBS and 0.055 mM 2-mercaptoethanol (Gibco, # 21985023). To differentiate THP-1 monocytes into macrophages, cells were treated with 100 nM PMA (Sigma, # P1585) for 48 h, followed by 24 h recovery in PMA-free medium. Primary BP tumor cells were derived from mouse BP mammary tumors. Briefly, single-cell suspensions obtained from dissociated tumors were grown in serum-free F-Medium [1:3 mixture of Ham’s F-12 and DMEM (Gibco) supplemented with 25 ng/mL hydrocortisone (Sigma), 5 μg/mL insulin (Thermo Fisher), 8.5 ng/mL cholera toxin (Sigma), 0.125 ng/mL EGF (Sigma), 100 μg/mL penicillin–streptomycin (Gibco) and 5 μM of the Rock1 inhibitor Y-27632 (Selleck)]. After tumor cell selection, tumor cells were maintained in F-Medium supplemented with 10% FBS. BRCA1-reconstituted MDA-MB-436, HCC1937 and BP cells were generated using pBABE-puro HA-BRCA1, a gift from Stephen Elledge (Addgene, # 14999)^71^.

Mouse macrophages were derived from the bone marrow (BM) of FVB/N mice by modifying previously described protocols^72^. Briefly, BM cells were seeded on ultra-low attachment plates (Corning) or Petri dishes (Falcon) and cultured in DMEM growth medium (DMEM + 10% FBS + 100 μg/mL penicillin–streptomycin) supplemented with 10 ng/mL mouse M-CSF (BioLegend, # 576404). On day 3, cell culture was top-up with fresh DMEM growth medium (same as original volume) with 10 ng/mL M-CSF. Cells were then incubated for another 4 days before harvesting adherent BM-derived macrophages (BMDMs).

For preparation of tumor cell-conditioned media (CM), tumor cells were grown to 60% of confluence, washed twice with PBS, and then incubated with fresh DMEM with or without 5 μM olaparib for two days. CM were then harvested and centrifuged to collect the supernatant.

### Tumor growth and treatment

Mammary tumor cells for orthotopic injection were resuspended in serum-free DMEM containing 40% matrigel (Corning) and injected orthotopically into the mammary fat pads of 6–8-week-old mice. 5 × 10^5^ BP tumor cells in a total volume of 100 μL were injected into mammary fat pads of female FVB/N mice (The Jackson Laboratory, # 001800). 1 × 10^5^ EO771-sgBRCA1 or EO771-sgBRCA1-sgSTING tumor cells in 100 μL were injected into mammary fat pads of female C57BL/6J (The Jackson Laboratory, # 000664) or *Tmem173*^gt/gt^ C57BL/6J mice (The Jackson Laboratory, # 017537).

Tumor growth was examined by measuring the greatest longitudinal diameter (length) and the greatest transverse diameter (width) with digital calipers, and tumor volume was calculated by use of the modified ellipsoid formula (0.52 × length × width^2^). Tumors were measured 2–3 times a week. All tumor measurements within single cohorts were performed by the same researcher. Tumor-bearing mice were randomized prior to start of treatment. Drug treatments were started when mean tumor volumes approximated 50–100 mm^3^. Mice were euthanized by CO_2_ inhalation when tumor volumes met humane endpoints described in the IACUC protocols or upon severe health deterioration. The maximum tumor diameter permitted under the relevant animal protocols is 20 mm, and this limit was not exceeded in any experiment.

Olaparib (MedChem, # HY-10162) was prepared by diluting 100 mg/mL stocks in DMSO with 10% (2-Hydroxypropyl)-β-cyclodextrin (HPCD, MedChem, # 101103) in PBS and administered immediately after drug preparation by intraperitoneal (IP) injection at a dose of 50 mg/kg body weight daily. ADU-S100 (MedChem, # HY-12885B) was reconstituted at 10 mM in DMSO. DMXAA (Sigma, # D5817) was reconstituted at 10 mg/mL in 7.5% NaHCO_3_ (Gibco). For intratumoral (IT) injection, 250 μg DMXAA (approximately 10 mg/kg body weight) was administered once weekly for a total of 3 doses. For IP injections, DMXAA was administered at a dose of 10 mg/kg body weight once weekly and terminated if tumor size exceeded 600 mm^3^. Anti-mouse PD-1 antibody (clone 332.8H3, kindly provided by Dr. Gordon Freeman at DFCI)^73^ was injected by IP at a dose of 10 mg/kg every 3 days. Anti-mouse CSF1R neutralizing antibody (clone CD115; BioXcell, # BE0213) was dosed at 40 mg/kg via IP every 3 days. For IFNAR1 blockade, anti-mouse IFNAR1 neutralizing antibody (200 μg/mouse; clone MAR1-5A3; BioXcell, # BE0241) was administered via IP 72 h and 24 h before start of the combination therapy (olaparib + DMXAA) and every 3 days thereafter. For CD8^+^ T cell depletion, anti-mouse CD8α neutralizing antibody (400 μg/mouse; clone YTS 169.4; BioXcell, # BE0117) or rat IgG2b isotype control, anti-keyhole limpet hemocyanin (400 μg/mouse; clone LTF-2; BioXcell, # BE0090) was administered via IP 48 h and 24 h before the combination therapy (olaparib + DMXAA) and every 4 days thereafter.

### Tissue digestion

To obtain single-cell suspensions, tumors were excised, minced and dissociated in collagenase/hyaluronidase buffer [DMEM with 5% FBS, 10 mM HEPES (Gibco), 100 μg/mL penicillin–streptomycin, 20 μg/mL DNase I (StemCell) and 1× collagenase/hyaluronidase (StemCell)] for 45 min at 37 °C with agitation, followed by treatment with ammonium-chloride-potassium (ACK) buffer (Lonza) for red blood cell (RBC) lysis, and strained through a 70 μm strainer to remove undigested tumor tissues. Spleens and tumor-draining lymph nodes (TDLNs) were mechanically dissociated by passing the tissues through a 70 μm strainer using the plunger of a 5 mL syringe, and RBCs were lysed as described above.

### Flow cytometry

For flow cytometry analyses, single-cell suspensions were obtained as described above (see the section “Tissue digestion”). Cells were stained in cold FACS buffer (PBS containing 0.2% BSA and 5 mM EDTA) with LIVE/DEAD Fixable Aqua Dead Cell Stain (Thermo Fisher) for 30 min on ice, followed by blocking with anti-mouse CD16/32 (1:50, 93, BioLegend) for 20 min on ice. Cells were then incubated in FACS buffer for 30 min on ice with antibodies specific to CD45 (1:100, 30-F11, BioLegend), TCR β chain (1:100, H57-597, BioLegend), CD3ε (1:100, 145-2C11, BioLegend), CD4 (1:100, RM4-5, BioLegend), CD8a (1:100, 53-6.7, BioLegend), CD44 (1:100, IM7, BioLegend), CD62L (1:100, MEL-14, BioLegend), CD25 (1:100, PC61, BioLegend), IFN-γ (1:100, XMG1.2, BioLegend), TNF-α (1:100, MP6-XT22, BioLegend), Granzyme B (1:100, NGZB, eBioscience), CD11c (1:100, N418, BioLegend), I-A/I-E (1:100, M5/114.15.2, BioLegend), CD86 (1:100, GL-1, BioLegend), CD11b (1:100, M1/70, BioLegend), F4/80 (1:100, BM8, BioLegend), CD206 (1:100, MMR, BioLegend), phospho-TBK1 (Ser172) (1:50, D52C2, Cell Signaling Technology) or phospho-IRF-3 (Ser396) (1:50, D6O1M, Cell Signaling Technology). For intracellular staining, Foxp3/Transcription Factor Staining Buffer Set (eBioscience, # 00-5523-00) was used for fixation and permeabilization. For cytokine analysis, cells were stimulated with Leukocyte Activation Cocktail with protein transport inhibitor Brefeldin A (BD Biosciences, # 550583) at 37 °C for 4 h prior to the staining. Analysis of human macrophages was performed by using human TruStain FcX (1:20, BioLegend), CD45 (1:20, HI30, BioLegend), CD11b (1:100, M1/70, BioLegend), CD163 (1:20, GHZ/61, BioLegend), HLA-DR (1:20, L243, BioLegend) and CD86 (1:100, IT2.2, BioLegend). Analysis of p-H2AX (Ser139) was performed according to manufacturer’s instructions. Briefly, ice-cold 70% ethanol (Decon) was added dropwise to the cell pellet while vortexing. Cells were then incubated at −20 °C for 1 h and washed three times with cold staining buffer. For the staining, 5 μL p-H2AX antibody (1:20, clone 2F3, BioLegend) was added to approximately 1 × 10^6^ cells in 100 μL staining buffer and incubated at 4 °C for 30 min. For Annexin V and 7-AAD staining, cells were detached with accutase (Sigma, # A6964). The detached cells (from culture medium and accutase treatment) were washed twice with cold PBS, and then incubated with 5 μL FITC Annexin V (BioLegend, # 640906) and 10 μL 7-AAD (BioLegend, # 420404) in 100 μL Annexin V binding buffer (BioLegend, # 422201) at room temperature for 15 min. Flow cytometry was performed on an LSR Fortessa HTS analyzer (BD Biosciences). Data were collected using BD FACSDiva (version 6) and analyzed using FlowJo (version 10.4). Gating strategies are shown in Supplementary Fig. 7.

### Clonogenic assay

Cells were filtered through a 40 μm mesh strainer (Corning), plated at a density of 5000 cells per well in a six-well plate and allowed to adhere overnight. Olaparib treatment was started on the next day. After 5 days, cells were fixed and stained with crystal violet (Sigma). The plates were imaged with a flatbed scanner. Crystal violet staining was then quantified by solubilization with 10% acetic acid, with absorbance measured at 590 nm with 750 nm as a reference.

### Cell viability assay

Tumor cells were seeded in 96-well plates at a density of 5000 cells per well and allowed to adhere overnight. Cells were then treated for 3 days with indicated drugs at the concentrations shown. Cell viability was measured using CellTiter-Glo 2.0 cell viability assay (Promega, #G9242) according to the manufacturer’s instruction. Growth inhibition was calculated by comparing the absorbance at 490 nm of drug-treated wells to that of untreated controls set at 100%. Dose–response curves and IC_50_ values were generated using a non-linear regression model in GraphPad Prism 9.

### Co-culture experiments

For in vitro co-culture of CD8^+^ T cells and macrophages, TAMs (7-AAD^−^CD45^+^CD11b^+^F4/80^+^) were isolated from BP tumors 14 days after transplantation of tumor cells using a FACSAria II cell sorter (BD Biosciences), TEMs were derived by incubating BMDMs of FVB/NJ mice with 50% BP-CM for 2 days, and CD8^+^ T cells were isolated from spleens of FVB/NJ mice using a mouse CD8^+^ T cell isolation kit (StemCell, # 19853). In a 96-well plate, 1 × 10^5^ CD8^+^ T cells per well were cultured alone or co-cultured with TAMs or TEMs at a ratio of 1:1 in RPMI 1640 supplemented with 10% FBS, 0.055 mM 2-mercaptoethanol, 2 ng/mL IL-2 (Peprotech), 2.5 ng/mL IL-7 (Peprotech) and 50 ng/mL IL-15 (Peprotech) for 2 days. For in vitro co-culture of tumor cells with macrophages, 2 × 10^5^ tumor cells per well were co-cultured with macrophages at a ratio of 1:1 (BP cells: mouse BMDMs), 1:2 (MDA-MB-436 cells: THP-1 macrophages), or 1:2 (HCC1937 cells: THP-1 macrophages) in six-well plates with indicated drug treatments or DMSO vehicle control for two days.

### Generation of STING-deficient cells and BRCA1-deficient cells

CRISPR Double Nickase Plasmids with improved editing specificity^74^ were used to generate BRCA1-deficient EO771 cells (EO771-sgBRCA1) and STING-deficient cells (BP-sgSTING and EO771-sgBRCA1-sgSTING). Briefly, tumor cells cultured in six-well plates were transfected with 2 μg/well of *Tmem173* (STING) double nickase plasmid (Santa Cruz, # SC-428364-NIC), *Brca1* double nickase plasmid (Santa Cruz, # SC-419362-NIC), or control double nickase plasmid (Santa Cruz, # SC-437281) using lipofectamine 3000 (Invitrogen). Two days after transfection, cells were screened for GFP-positive cells and cultured in growth medium containing puromycin (Thermo Fisher) for selection.

### RNA extraction and reverse transcription-quantitative PCR (RT-qPCR)

Total RNA was extracted using RNeasy Plus Mini Kit (QIAGEN, # 74134). An iScript reverse transcription supermix (Bio-Rad, # 1708841) was used for the first-strand cDNA synthesis with 1 μg total RNA. Real-time PCR was performed using SYBR™ Select Master Mix (Thermo Fisher, #4472908) with gene-specific primers (mouse *Il6*, forward 5′-TAGTCCTTCCTACCCCAATTTCC-3′, reverse 5′-TTGGTCCTTAGCCACTCCTTC-3′; mouse *Il1b*, forward 5′-GCAACTGTTCCTGAACTCAACT-3′, reverse 5′-ATCTTTTGGGGTCCGTCAACT-3′; mouse *Cxcl1*, forward 5′-CCGAAGTCATAGCCACACTCAA-3′, reverse 5′-GCAGTCTGTCTTCTTTCTCCGTTAC-3′; mouse *Ifnb*, forward 5′-TCCGAGCAGAGATCTTCAGGAA-3′, reverse 5′-TGCAACCACCACTCATTCTGAG-3′; mouse *Ccl5*, forward 5′-GCTGCTTTGCCTACCTCTCC-3′, reverse 5′-TCGAGTGACAAACACGACTGC-3′; mouse *Cxcl10*, forward 5′-CCAAGTGCTGCCGTCATTTTC-3′, reverse 5′-GGCTCGCAGGGATGATTTCAA-3′; mouse *Actb*, forward 5′-CGGTTCCGATGCCCTGAGGCTCTT-3′, reverse 5′-CGTCACACTTCATGATGGAATTGA-3′; human *IL6*, forward 5′-ACTCACCTCTTCAGAACGAATTG-3′, reverse 5′-CCATCTTTGGAAGGTTCAGGTTG-3′; human *IL1B*, forward 5′-ATGATGGCTTATTACAGTGGCAA-3′, reverse 5′-GTCGGAGATTCGTAGCTGGA-3′; human *CXCL1*, forward 5′-AAGTGTGAACGTGAAGTCC-3′, reverse 5′-GGATTTGTCACTGTTCAGCA-3′; human *GAPDH*, forward 5′-CTCTGCTCCTCCTGTTCGAC-3′, reverse 5′-TTAAAAGCAGCCCTGGTGAC-3′). Relative mRNA levels were calculated using the ∆∆C_T_ method. Mouse *Actb* and human *GAPDH* were used as endogenous controls for mouse and human samples, respectively.

### Immunohistochemistry

Tumor fragments were fixed in 10% formalin overnight and transferred to 70% ethanol. Embedding, sectioning and H&E staining were then performed by Harvard rodent histopathology core. Histological characteristics of BP tumors were examined by independent pathologists at Harvard medical school. IHC staining was performed as described previously^75^. Antibodies used for IHC staining include anti-BRCA1 antibody (1:1200, Abcam, # ab238983), anti-ERα antibody (1:200, clone SP1, Fisher, # RM9101S0), anti-Ki67 antibody (1:500, Abcam, # ab15580), and anti-HER2 (1:500, clone 29D8, Cell Signaling, # 2165).

### Tissue cyclic immunofluorescence (CyCIF) analysis

CyCIF was used to identify immune cell subsets as previously described^76,77^. Additional details and methods are available at www.cycif.org. Briefly, formalin fixed paraffin-embedded tumor tissues were cut into 5 μm-thick sections, which underwent automated dewaxing and heated antigen retrieval using BOND autostainer (Leica). Tissues then underwent iterative staining, imaging and bleaching with antibodies specific to Pan-Cytokeratin (1:100, AE1/AE3, eBioscience), CD45 (1:100, 30-F11, BioLegend), F4/80 (1:100, BM8, BioLegend), CD11b (1:100, EPR1344, Abcam), CD11c (1:100, D1V9Y, Cell Signaling Technology), CD8a (1:300, 4SM16, eBioscience), CD4 (1:100, 4SM95, eBioscience). Imaging was performed on InCell Analyzer 6000 (GE) at 20× with uniform exposure for each channel. Images underwent stitching, registration, BaSIC correction using mcmicro (www.mcmicro.org)^78^. Quantification was performed in R and GraphPad Prism 9. Raw fluorescence intensity data was first normalized across all samples. Thresholds were determined by fitting a two-Gaussian mixture model to the normalized intensity data and the positive threshold was chosen to be peak of the positive distribution. Normalized values were then mapped back to raw values for calculation. Tumor and immune cell subsets were identified using uniform marker thresholds. Iterative gates were used to select multi-marker cell types. Cell typing was confirmed by random sampling of images and visual confirmation.

### Double-stranded DNA (dsDNA) staining

For the dsDNA staining of cultured cells, *BRCA1*-deficient tumor cells cultured on glass coverslips in six-well plates were treated with 5 μM olaparib or DMSO vehicle control for 2 days in the presence or absence of macrophage-derived CM. Following the treatment, cells were fixed and stained for dsDNA fragments in the cytosol as previously described^79^. Briefly, cells were first fixed with 4% paraformaldehyde for 10 min. Selective plasma membrane permeabilization was then performed by incubating the fixed cells with 0.02% saponin (Sigma) in PBS for 5 min. Cells were blocked with 2.5% normal goat serum in PBS for 30 min and stained with an anti-dsDNA antibody (1:200 dilution, clone AE-2, Sigma, # MAB1293) in PBS with 1% BSA at 4 °C overnight, followed by staining with goat anti-mouse IgG (H + L) highly cross-adsorbed secondary antibody, Alexa Fluor 594 (1:400 dilution, Thermo Fisher, # A-11032) alone or together with an anti-CD11b antibody, Alexa Fluor 488 (1:100 dilution, clone M1/70, BioLegend, # 101219). Cells were mounted with ProLong Diamond Antifade Mountant with DAPI (Thermo Fisher, # P36966). For the dsDNA staining of tumor tissues, samples were snap frozen and embedded in O.C.T. Compound (Sakura). Frozen sections were fixed and stained for dsDNA as described above. Tissues were also stained using a pan-Cytokeratin antibody, Alexa Fluor 488 (1:50 dilution, clone C11, Santa Cruz, # sc-8018). Staining was imaged using a Leica SP5X laser scanning confocal microscope or a BioTek Cytation 5 cell imaging multi-mode reader. Fluorescence intensity of dsDNA fragments in the cytosol was analyzed using ImageJ/Fiji as previously described^17^.

### Immunoblotting

Cells were pelleted and lysed using ice-cold RIPA buffer supplemented with protease and phosphatase inhibitor cocktail (Thermo Fisher). Protein concentration was determined using DC protein assay (Bio-Rad). Equal amounts of protein extracts (40–60 μg) were loaded and separated by SDS–PAGE, and then transferred to polyvinylidene fluoride (PVDF) membranes. Membranes were blocked for 45 min at room temperature with 5% non-fat milk (Bio-Rad) in TBS plus 0.05% Tween 20, followed by overnight incubation at 4 °C with primary antibodies against BRCA1 (1:500, Abcam, # ab238983), STING (1:1000, clone D2P2F, Cell Signaling Technology, # 13647S), or VINCULIN (1:2000, clone hVIN-1, Sigma, # V9131). Blots were then incubated with fluorescently labeled anti-mouse IgG (1:2000, Rockland Immunochemicals, # RL610-145-002) or anti-rabbit IgG (1:2000, Molecular Probes, # A-21109) at room temperature for 1 h. Western blots were visualized on an Odyssey scanner (LI-COR). Uncropped blots are provided in the Source Data file.

### IFNβ ELISA

Tumor cells were treated with olaparib or DMSO vehicle control for 2 days. Cell culture supernatants were then harvested and subjected to centrifugation at 1500 × *g* for 10 min at 4 °C to remove floating cells and debris. IFNβ was detected via mouse IFNβ ELISA Kit (Thermo Fisher, # 424001) according to manufacturer’s instructions.

### Analysis of the cancer genome atlas (TCGA) and gene expression omnibus (GEO) data

For the TCGA cohort, RNA-seq data of breast and ovarian cancers were obtained from a GEO dataset (GEO: GSE62944), where the TCGA RNA-seq data of different cancer types were re-processed by aligning the FASTQ files downloaded from the Cancer Genomics Hub so that the gene expression could be compared across cancer types^80^. *BRCA1* mutation information of patients in TCGA cohort were retrieved according to a recent study^81^. In addition, another two datasets from GEO database (GSE27830, breast cancer; GSE63885, ovarian cancer) with whole exome transcriptome detected with the same platform (GPL570) were also recruited and analyzed together as the GEO cohort. Raw CEL files of these two datasets obtained from GeneChip Human Genome U133 Plus 2.0 Array were downloaded from GEO database and then processed together using R package ‘affy’ (version 1.64.0) so that they could be combined for further analysis. We normalized and annotated all the CEL files using robust multi-array average (RMA) algorithm and corresponding annotation files from R Bioconductor (version 3.10) to obtain summarized values for each probeset. Multiple probesets corresponding to a single gene were summarized into a gene symbol by taking the probeset with highest mean value. *BRCA1* mutation status for each case in these two datasets were acquired from clinical information of the corresponding studies, which were also archived in GEO (https://www.ncbi.nlm.nih.gov/geo/). M2 TAM immunosuppressive gene signature was derived from a previous study^30^. Enrichment scores of M2 signature were generated by single-sample gene set enrichment analysis (ssGSEA), using R package ‘gene set variation analysis (GSVA)’ (version 1.34.0). We also carried out GSEA (version 4.0.3) analysis using MSigDB v7.2 HALLMARK, AZARE and Reactome gene sets. For GSEA, genes were first ranked according to log_2_(fold change), and then analyzed using GSEAPreranked tool (v6.0.12) with the ‘classic’ method^82^.

### Transcriptome analysis

Total RNA was isolated by RNeasy Plus Mini Kit (QIAGEN) and sequenced on an Ion Torrent platform (Thermo Fisher) using an Ion AmpliSeq Custom Panel targeting 4604 murine genes most relevant to our studies, as we have previously described^16,83^. To generate read counts per gene, data were analyzed using Torrent Suite and AmpliSeqRNA analysis plugin (Thermo Fisher). We then studied differential gene expression using DESeq2 package (version 1.26.0)^84^ in R software environment (version 3.6.3). Genes with log_2_(fold change) >1 and *P* < 0.001 were considered differentially expressed genes (DEGs). Volcano plots showing the significance and magnitude of log_2_(fold change) of these DEGs were generated by ‘ggplot2’ package (version 3.3.5) in R. Gene ontology (GO) analysis of DEGs was performed using R package ‘ClusterProfiler’ (version 3.14.3). GSEA analysis was performed as described above. Heat maps illustrating changes in gene expression were generated using the heatmap3 function of ‘gplots’ package (version 3.1.1) in R.

### Statistical analyses

Statistical analyses were performed with Prism 9.3.1 (GraphPad Software Inc.). Two-way ANOVA with Tukey’s multiple comparisons test was used for tumor growth analysis. Log-rank Mantel–Cox test was used for survival analysis. For other analyses, unpaired two-tailed Student’s *t* test (for normally distributed data) and Mann–Whitney nonparametric test (for skewed data that deviate from normality) were used to compare two conditions. One-way ANOVA with Tukey’s multiple comparisons test (for normally distributed data) and Kruskal–Wallis nonparametric test (for skewed data) were used to compare three or more means. Differences with *P* < 0.05 were considered statistically significant.

### Reporting summary

Further information on research design is available in the Nature Research Reporting Summary linked to this article.

## Supplementary information

Supplementary Information

Reporting Summary

## Data Availability

Transcriptomic data generated in this study have been deposited in Gene Expression Omnibus (GEO) under accession number GSE201017. Publicly available datasets used in this study are available in GEO under accession numbers GSE62944, GSE63885, and GSE27830. *BRCA1* mutation data for the TCGA cohort were downloaded from ref. ^81^. *BRCA1* mutation data for the GSE63885 and GSE27830 cohorts were retrieved from the corresponding clinical data stored in the GEO database. The remaining data are available within the Article, Supplementary Information or Source Data file. Source data are provided with this paper.

## Source data

Source Data
