# Supplementary material for: STING agonism reprograms tumor-associated macrophages and overcomes resistance to PARP inhibition in BRCA1-deficient models of breast cancer
Source: Nat Commun. 2022 May 31;13:3022. doi: 10.1038/s41467-022-30568-1 (PMC9156717; doi:10.1038/s41467-022-30568-1)

**Supplementary Information**

# STING Agonism Reprograms Tumor-associated Macrophages and Overcomes Resistance to PARP inhibition in BRCA1-deficient Models of Breast Cancer

Qiwei Wang[1,2,3,11], Johann S. Bergholz[1,2,3,11], Liya Ding[1,3,4], Ziying Lin[1,2,5], Sheheryar K. Kabraji[1,6,7], Melissa E. Hughes[6], Xiadi He[1,2], Shaozhen Xie[1,2], Tao Jiang[1], Weihua Wang[1], Jason J. Zoeller[8], Hye-Jung Kim[9], Thomas M. Roberts[1,2], Panagiotis A. Konstantinopoulos[6], Ursula A. Matulonis[6], Deborah A. Dillon[10], Eric P. Winer[6], Nancy U. Lin[6], Jean J. Zhao[1,2,3,7*]

[1] Department of Cancer Biology, Dana-Farber Cancer Institute, Boston, MA, USA

[2] Department of Biological Chemistry and Molecular Pharmacology, Harvard Medical School, Boston, MA, USA

[3] Broad Institute of Harvard and MIT, Cambridge, MA, USA

[4] Department of Medicine, Harvard Medical School, Boston, MA, USA

[5] Department of Respiratory and Critical Care Medicine, The First Affiliated Hospital of Sun Yat-sen University, Guangzhou, Guangdong, China

[6] Department of Medical Oncology, Dana-Farber Cancer Institute, Boston, MA, USA

[7] Laboratory of Systems Pharmacology, Harvard Medical School, Boston, MA, USA

[8] Department of Cell Biology and Ludwig Center at Harvard, Harvard Medical School, Boston, MA, USA

[9] Department of Cancer Immunology and Virology, Dana-Farber Cancer Institute, Boston, MA, USA

[10] Department of Pathology, Brigham and Women's Hospital, Boston, MA, USA

[11] These authors contributed equally to this work

*Correspondence and requests for materials should be addressed to
J.J.Z. (jean_zhao@dfci.harvard.edu)

**Supplementary Figure. 1. *Brca1$^{L/L}$Trp53$^{L/L}$* mouse model of mammary tumors induced by intra-ductal injection of Ade-Cre.**

**(a) Left**, RT-qPCR analysis of *Brca1* in mouse mammary epithelial cells (MMECs) and *Brca1$^{-/-}$ Trp53$^{-/-}$* (BP) mouse mammary tumor cells (n = 3). **Right**, RNA-seq analysis of *Trp53* in BP and MMECs (CPM, counts per million mapped reads; MMEC, n = 5; BP, n = 3).

**(b-c)** Representative staining of normal mouse mammary tissue (FVB strain, one mouse) and BP mouse mammary tumors (FVB strain, two mice). Scale bars, 100 μm.

**(d)** Representative H&E staining of BP mammary tumors from three mice. Scale bar, 100 μm.

**(e)** Western blot of BP and BRCA1-reconstituted BP (BP+BRCA1) tumor cells. Representative blots of three independent experiments are shown.

**(f-g)** Clonogenic assay measuring the responses of BP and BP+BRCA1 tumor cells to olaparib. Representative images (**f**) and quantification of crystal violet staining (**g**, n = 3) are shown.

**(h)** CyCIF analysis of BP tumors. Representative images of two mice are shown on the left. Scale bars, 100 μm. Quantification of respective cell populations as a percentage of $CD45^+$ cells in the tumors are shown on the right. CD4, n = 6 fields; CD8, n = 7 fields; CD11b, n = 4 fields; F4/80, n = 3 fields; CD11c, n = 3 fields examined over two mice.

**(i)** Gating strategies for flow cytometry analyses described in Fig. 1d and Supplementary Fig. 1j and k. Flow cytometry plot axes are displayed in logarithmic scale, except for forward side scatter (FSC) for which a linear scale was used.

**(j)** Flow cytometry analysis of BP tumors from FVB mice with or without olaparib treatment, showing assessment of intratumoral $CD8^+$ T cells after seven days of treatment. Each dot represents data from a single tumor. Control, n = 6; Olaparib, n = 8.

**(k)** Analysis of tumors from FVB mice bearing *Brca1*-deficient mouse PBM ovarian tumors or *Brca1*-deficient mouse BP breast tumors 28 days after tumor cell implantation for TAMs (CD45$^{+}$ CD11b$^{+}$ F4/80$^{+}$), M1-like (CD206$^{-}$ MHC-I $^{high}$) and M2-like (CD206$^{+}$ MHC-II$^{lowa}$) TAM polarization phenotypes. Flow cytometry plot axes are displayed in logarithmic scale. n = 5 for each group. Each dot represents data from a single tumor.

**(l-m)** Analyses of M2 immunosuppressive gene signature.

**(l)** TCGA cohorts of patients with *BRCA1* mutant (mut) ovarian cancer (n = 29) and patients with *BRCA1* mut breast cancer (n = 30).

**(m)** GEO cohorts of patient gene expression data from GSE63885 (*BRCA1* mut ovarian cancer, n = 28) and GSE27830 (*BRCA1* mut breast cancer, n = 47) datasets with whole exome transcriptome analyzed with same platform (GPL570).

**(n-o)** Gene Set Enrichment Analysis (GSEA) of Hallmark gene sets, Azare STAT3 targets, and Reactome. Analysis of TCGA cohorts of patients with *BRCA1* mut breast cancer (n = 30) and *BRCA1* mut ovarian cancer (n = 29) (**n**). Analysis of GEO cohorts of patient gene expression data from GSE63885 (n = 28) and GSE27830 (n = 47) datasets with whole exome transcriptome analyzed with same platform (GPL570) (**o**). Gene sets with false discovery rate (FDR) < 0.25 and normalized enrichment score (NES) > 1 are shown.

Data are presented as mean ± SEM (**a**, **g** and **h**), mean ± SD (**l** and **m**), or median with quartiles (violin plots, **j** and **k**). Two-tailed unpaired t test (**j**-**m**). One-way ANOVA (**g** and **h**). ns, not significant. Source data are provided as a Source Data file.

# Supplementary Fig. 1

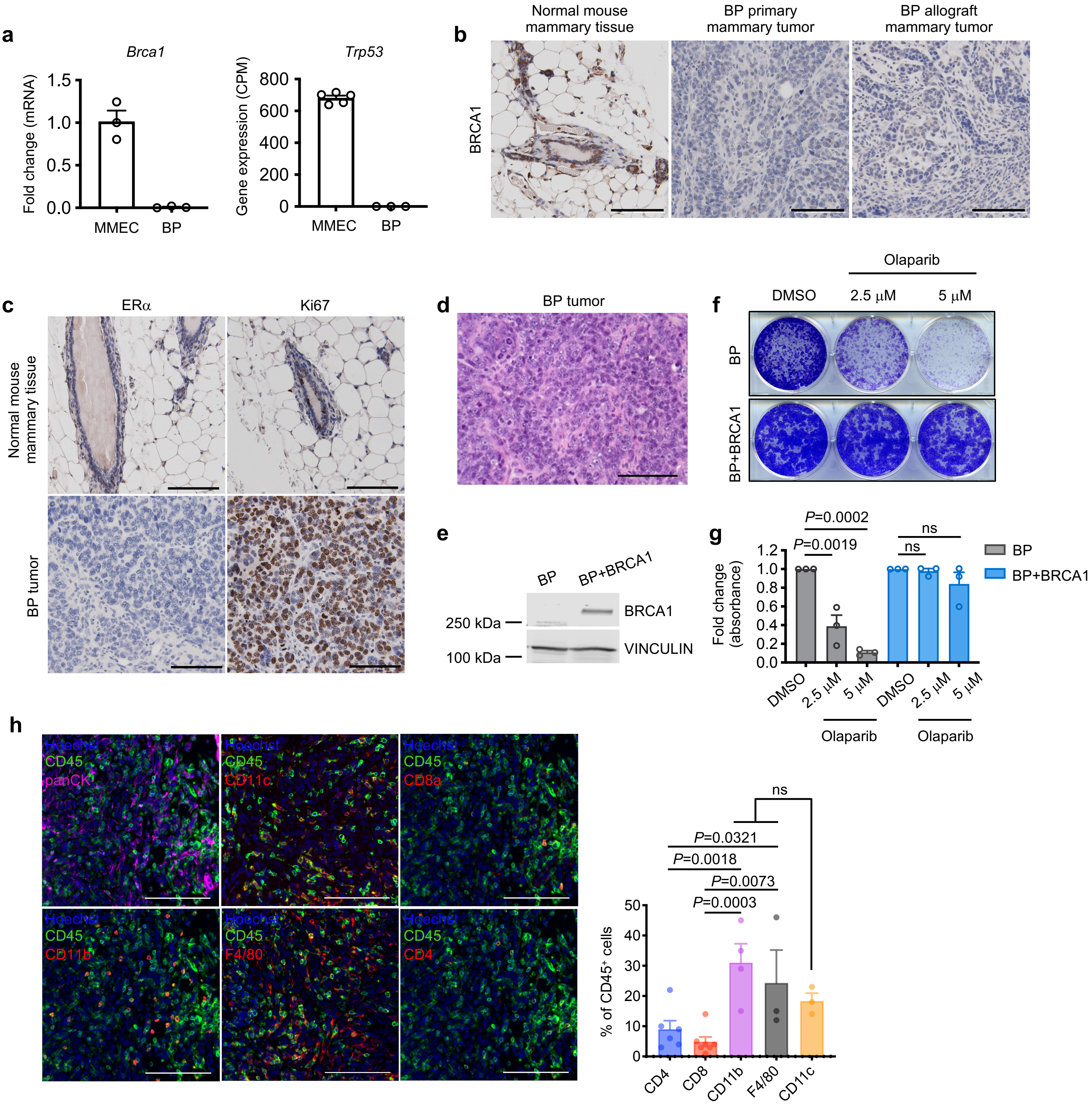

## Supplementary Fig. 1 (continued)

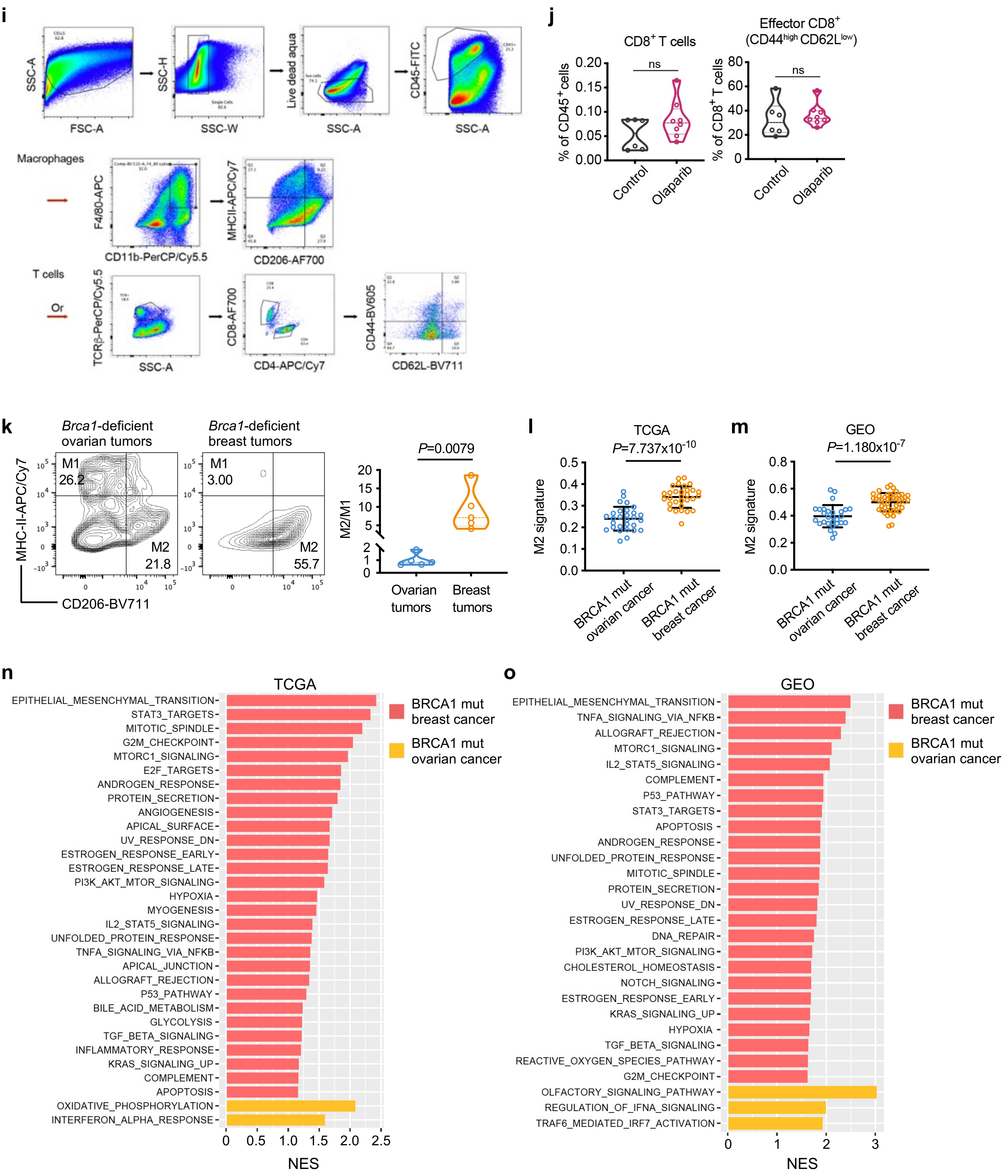

**Supplementary Figure. 2. *BRCA1*-deficient breast tumor cells induce pro-tumorigenic macrophage polarization *in vitro*.**

**(a-b)** Analysis of mouse bone marrow-derived macrophages (BMDMs, $CD11b^+$) co-cultured with BP tumor cells with or without olaparib (5 μM) treatment for five days (n = 4).

**(c)** Expression of CD163 and CD86 in THP-1 macrophages co-cultured with MDA-MB-436 human breast cancer cells with or without olaparib (5 μM) treatment for five days (THP-1, n = 4; THP-1/MDA-MB-436, n = 6).

**(d)** Analysis of mouse BMDMs co-cultured with PBM or BP cells for two days (n = 3).

**(e)** Analysis of mouse BMDMs co-cultured with BP cells or BP+BRCA1 cells for two days (n = 4).

**(f)** Western blot of MDA-MB-436 and BRCA1-reconstituted tumor cells. Representative blots of three independent experiments are shown.

**(g)** Expression of M2 (CD163) and M1 (CD86) markers in control THP-1 macrophages, or THP-1 macrophages co-cultured with MDA-MB-436 or MDA-MB-436+BRCA1 cells for two days (n = 4).

**(h)** Western blot of HCC1937 and BRCA1-reconstituted tumor cells. Representative blots of three independent experiments are shown.

**(i)** Expression of M2 (CD163) and M1 (CD86) markers in control THP-1 macrophages, or THP-1 macrophages co-cultured with HCC1937 or HCC1937+BRCA1 cells for two days (n = 4).

**(j)** Analysis of mouse BMDMs incubated with control medium or 50% conditioned media (CM) derived from BP, or olaparib-treated BP (BP/OL) tumor cells for two days (control medium, n = 6; BP-CM, n = 7; BP/OL-CM, n = 4).

**(k)** Analysis of cytokine production by $CD8^+$ T cells co-cultured with or without control mouse BMDMs or BP tumor cell-educated macrophages (TEMs) for two days (n = 4).

Data are presented as mean ± SEM. One-way ANOVA. ns, not significant. Flow cytometry plot axes are displayed in logarithmic scale. Source data are provided as a Source Data file.

**Supplementary Fig. 2**

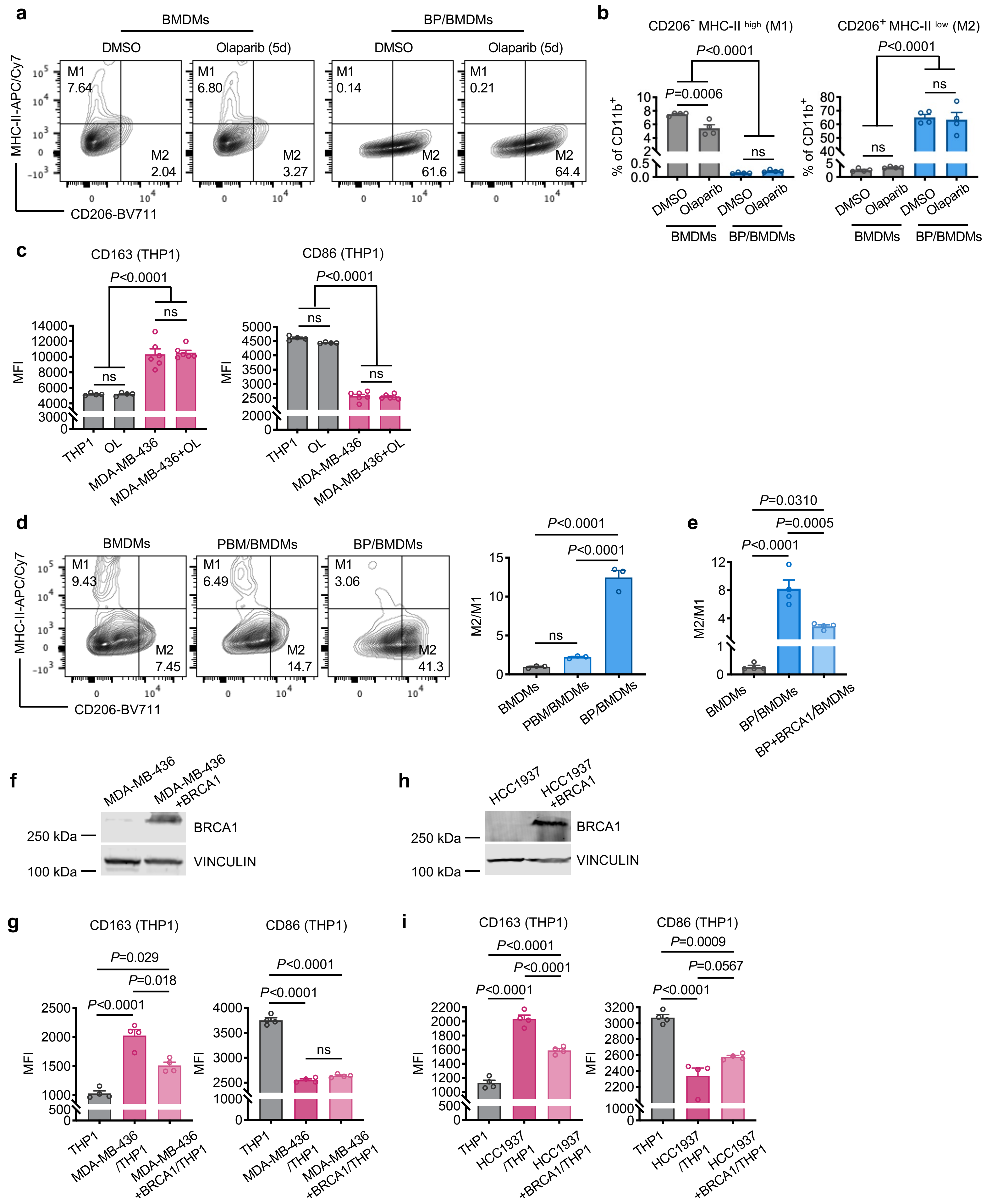

**Supplementary Fig. 2 (continued)**

j

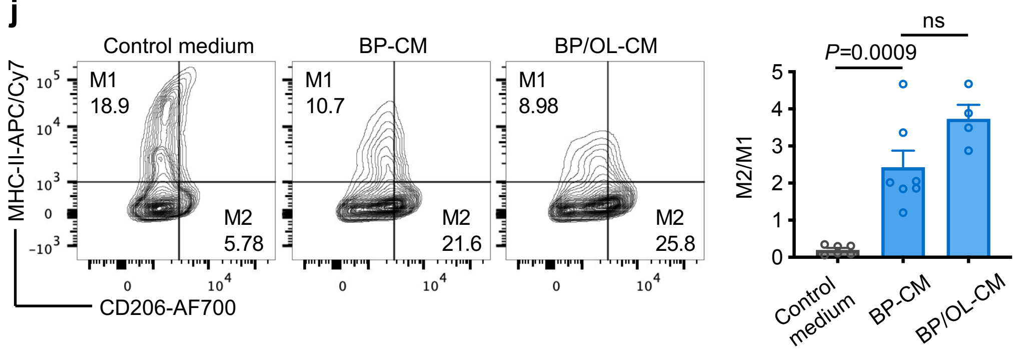


k

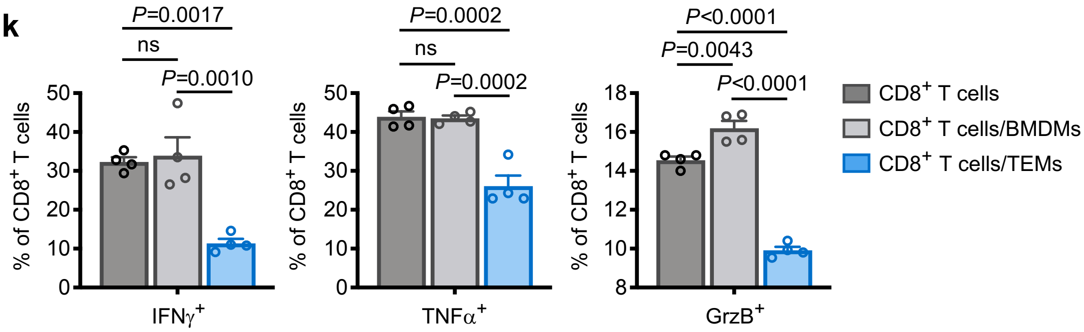

**Supplementary Figure. 3. Tumor cell-educated macrophages (TEMs) suppress synthetic lethal response of *BRCA1*-deficient breast tumor cells to olaparib.**

**(a)** Tumor cell (MDA-MB-436)-educated THP-1 macrophage (MDA-MB-436 TEMs)-derived conditioned medium (CM) was added to MDA-MB-436 cells, followed by two days of olaparib (OL, 5 μM) treatment. Cells were then stained using DAPI and an anti-dsDNA antibody. The intensity of dsDNA fragments in the cytosol was quantified. Scale bar, 100 μm. Control + DMSO, n = 10 fields; Control + OL, n = 10 fields; TEMs + DMSO, n = 6 fields; TEMs + OL, n = 6 fields examined over two independent experiments.

**(b-c)** Analysis of BP cells incubated with or without CM from control macrophages (Mφ) or BP TEMs followed by 16 hours of treatment with olaparib (5 μM). Cells were analyzed by flow cytometry for the percentage of cells positive for phosphorylation of histone H2AX at Ser139 (n = 3).

**(d)** Gating strategies and representative plots for flow cytometry analysis described in Fig. 3d. Flow cytometry plot axes are displayed in logarithmic scale.

**(e-h)** Tumor cells were incubated with or without TEM-CM or fractionated TEM-CM, followed by olaparib (5 μM) treatment. BP (**e** and **f**) and MDA-MB-436 (**g** and **h**) cells were analyzed for DNA damage response by staining for Ser139-phosphorylated H2AX (n = 3) and apoptosis (Annexin $V^{+}$ 7-$AAD^{-}$) [Control, n = 6; Olaparib, n = 6; TEM-CM + Olaparib, n = 3; TEM-CM (> 3 kDa) + Olaparib, n = 6; TEM-CM (< 3 kDa) + Olaparib, n = 6].

**(i)** BP cells were co-cultured with or without TAMs sorted from BP tumors, followed by two days of olaparib (OL, 5 μM) treatment. Cells in the co-culture were stained for CD11b and dsDNA. BP cells ($CD11b^{-}$) were analyzed for the intensity of cytoplasmic dsDNA. Scale bar, 100 μm. Control

+ DMSO, n = 8 fields; Control + OL, n = 8 fields; TAMs + DMSO, n = 8 fields; TAMs + OL, n = 8 fields examined over two independent experiments.

**(j)** Analysis of cytosolic dsDNA in BP tumor cells [pan-cytokeratin $(CK)^{+}$] *in vivo* after two i.p. injections of anti-CSF1R antibody and seven days of treatment with olaparib (OL) as monotherapy or in combination (schematic representation of this experiment is shown on the top right). Scale bar, 100 µm. Control, n = 5 fields; OL, n = 5 fields; anti-CSF1R, n = 5 fields; OL + anti-CSF1R, n = 5 fields examined over two mice for each group.

Data are presented as mean ± SEM. One-way analysis of variance (ANOVA). ns, not significant. Source data are provided as a Source Data file.

**Supplementary Fig. 3**

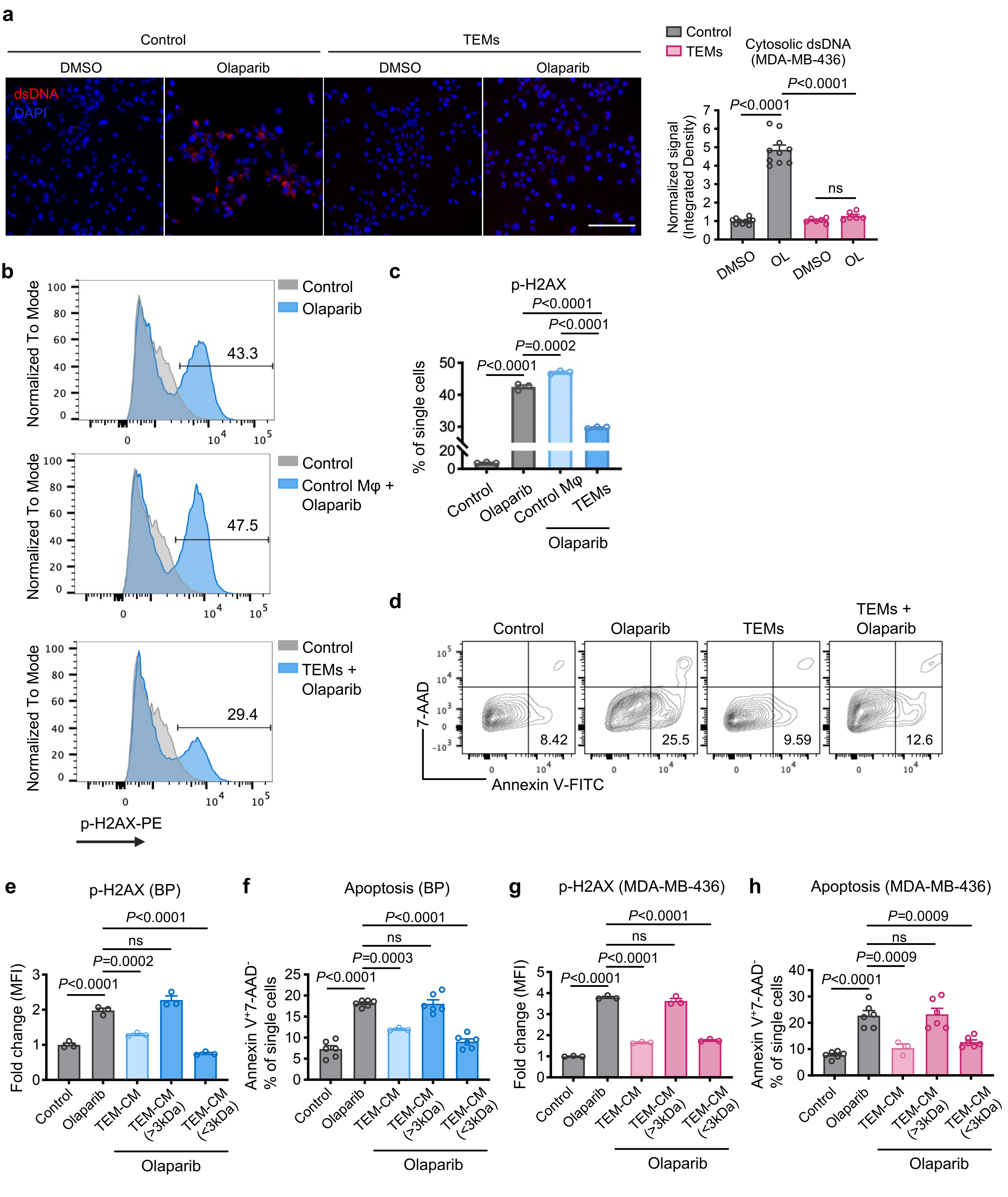

## Supplementary Fig. 3 (continued)

**i**

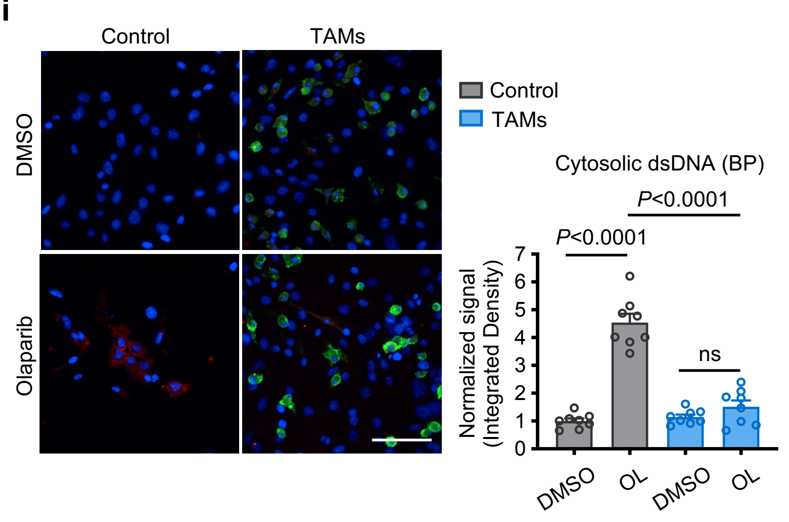


**j**

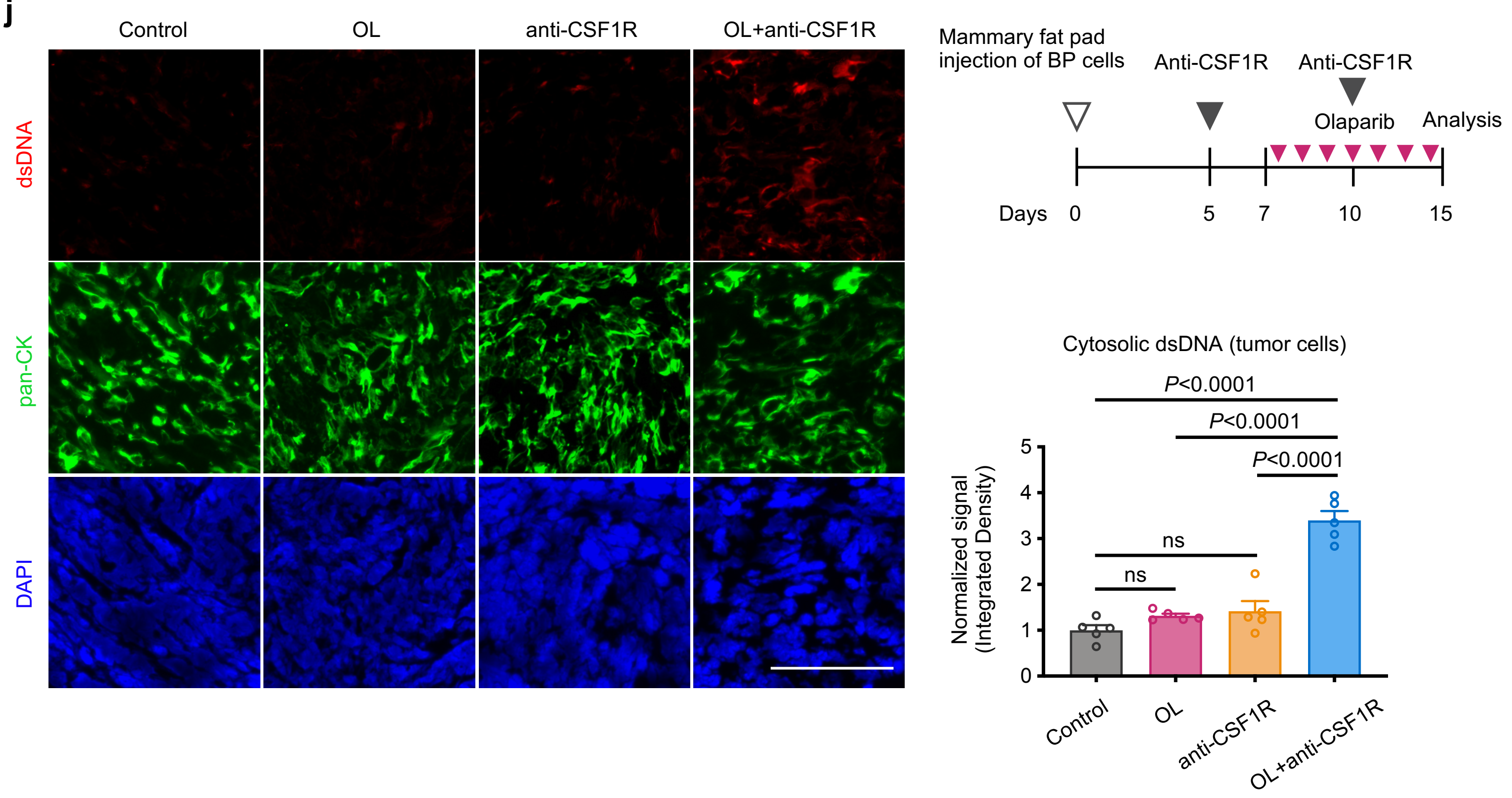

**Supplementary Fig. 4. STING agonists reprogram tumor cell-educated macrophages (TEMs) into an M1-like state *in vitro*.**

**(a)** Mouse BMDMs were subjected to transcriptome analysis after a 24-hour exposure to 50% conditioned media (CM) from olaparib-treated BP tumor cells (BP/OL) with or without DMXAA (0.05 mg/mL). n = 2 for each group. **Left**, volcano plot showing the significance and magnitude of changes in gene expression between macrophages treated with BP/OL-CM/DMXAA versus BP/OL-CM/DMSO. Statistical significance was calculated using a two-sided Wald test and adjusted for multiple testing using the Benjamini–Hochberg procedure. **Right**, top-ranked up-regulated and down-regulated gene ontology (GO) terms in macrophages treated with BP/OL-CM/DMXAA. Significance of enriched terms were adjusted using the Benjamini–Hochberg procedure for multi-testing.

**(b)** Images of mouse BMDMs after a 24-hour incubation with medium containing IL-4 (20 ng/mL), LPS (100 ng/mL), IFNγ (20 ng/mL), olaparib (5 μM), DMXAA (0.05 mg/mL), 50% BP-CM with or without DMXAA, or 50% BP/OL-CM with or without DMXAA. Images are representative of two independent experiments.

**(c)** Analysis of STING pathway activation (p-TBK1$^{+}$p-IRF3$^{+}$) of control BMDMs and BP TEMs treated with or without DMXAA for 24 hours (BMDMs + DMSO, n = 4; BMDMs + DMXAA, n = 4; TEMs+ DMSO, n = 3; TEMs+ DMXAA, n = 4).

**(d)** Images of γ-H2AX foci (green) and nuclei (DAPI, blue) in BP tumor cells treated with or without 5 μM olaparib for 1 day in the presence or absence of CM from naïve BMDMs (control Mφ), TEMs or DMXAA-treated TEMs. Scale bars, 100 μm. Images are representative of two independent experiments.

**(e)** Analysis of γ-H2AX foci shown in **(d)**. The percentage of cells with > 5 γH2AX foci per nucleus was quantified. Control, n = 13 fields; Olaparib, n = 13 fields; Control macrophages + Olaparib, n = 7 fields; TEMs + Olaparib, n = 13 fields; TEMs (DMXAA-treated) + Olaparib, n = 10 fields examined over two independent experiments.

Data are presented as mean ± SEM. One-way ANOVA (**c** and **e**). ns, not significant. Source data are provided as a Source Data file.

## Supplementary Fig. 4

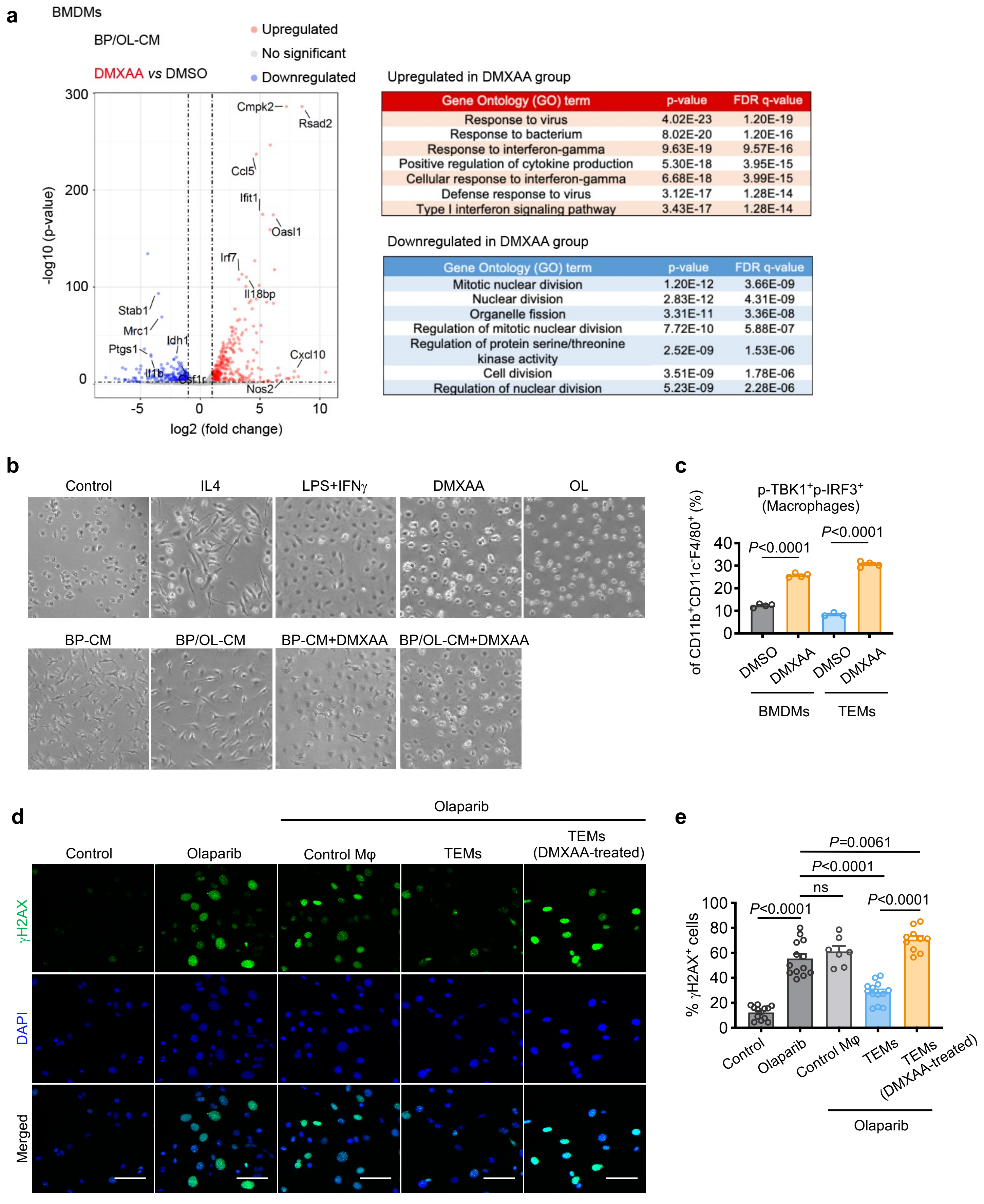


Upregulated in DMXAA group

| Gene Ontology (GO) term | p-value | FDR q-value |
|---|---|---|
| Response to virus | 4.02E-23 | 1.20E-19 |
| Response to bacterium | 8.02E-20 | 1.20E-16 |
| Response to interferon-gamma | 9.63E-19 | 9.57E-16 |
| Positive regulation of cytokine production | 5.30E-18 | 3.95E-15 |
| Cellular response to interferon-gamma | 6.68E-18 | 3.99E-15 |
| Defense response to virus | 3.12E-17 | 1.28E-14 |
| Type I interferon signaling pathway | 3.43E-17 | 1.28E-14 |

Downregulated in DMXAA group

| Gene Ontology (GO) term | p-value | FDR q-value |
|---|---|---|
| Mitotic nuclear division | 1.20E-12 | 3.66E-09 |
| Nuclear division | 2.83E-12 | 4.31E-09 |
| Organelle fission | 3.31E-11 | 3.36E-08 |
| Regulation of mitotic nuclear division | 7.72E-10 | 5.88E-07 |
| Regulation of protein serine/threonine kinase activity | 2.52E-09 | 1.53E-06 |
| Cell division | 3.51E-09 | 1.78E-06 |
| Regulation of nuclear division | 5.23E-09 | 2.28E-06 |

**Supplementary Fig. 5. Depletion of TAMs improves therapeutic response of orthotopic BP tumors to olaparib in syngeneic immunocompetent mice *in vivo*.**

**(a)** *Brca1*$^{-/-}$*Trp53*$^{-/-}$ (BP) tumor cells were transplanted into mammary fat pads of syngeneic FVB mice and treated with combination therapy of olaparib (OL) and intratumoral (IT) injection of DMXAA with or without anti-CD8 or an anti-CSF1R neutralizing antibodies (Control, n = 6; OL + DMXAA, n = 8; OL + DMXAA + anti-CSF1R, n = 8; OL + DMXAA + anti-CD8, n = 8). Tumor volumes were measured at day 23. Each dot represents data from a single tumor.

**(b)** Tumor growth of BP allografts in FVB mice treated with olaparib or anti-CSF1R as monotherapy or in combination. Control, n = 10; olaparib, n = 10; anti-CSF1R, n = 10; olaparib + anti-CSF1R, n = 10.

**(c-g)** Flow cytometry analysis of intratumoral immune cells from BP tumors after 21 days of treatment. Each dot represents data from a single tumor. (**c** and **d**) Control, n = 6; Olaparib, n = 8; aCSF1R, n = 9; Olaparib + aCSF1R, n = 10. (**e**) Control, n = 6; Olaparib, n = 6; aCSF1R, n = 6. (**f** and **g**) Control, n = 6; Olaparib, n = 6; DMXAA, n = 4.

Data are presented as mean ± SEM (**a** and **b**) or median with quartiles (violin plots, **c-g**). Two-way ANOVA (**b**). One-way ANOVA (**a** and **c-g**). ns, not significant. Source data are provided as a Source Data file.

**Supplementary Fig. 5**

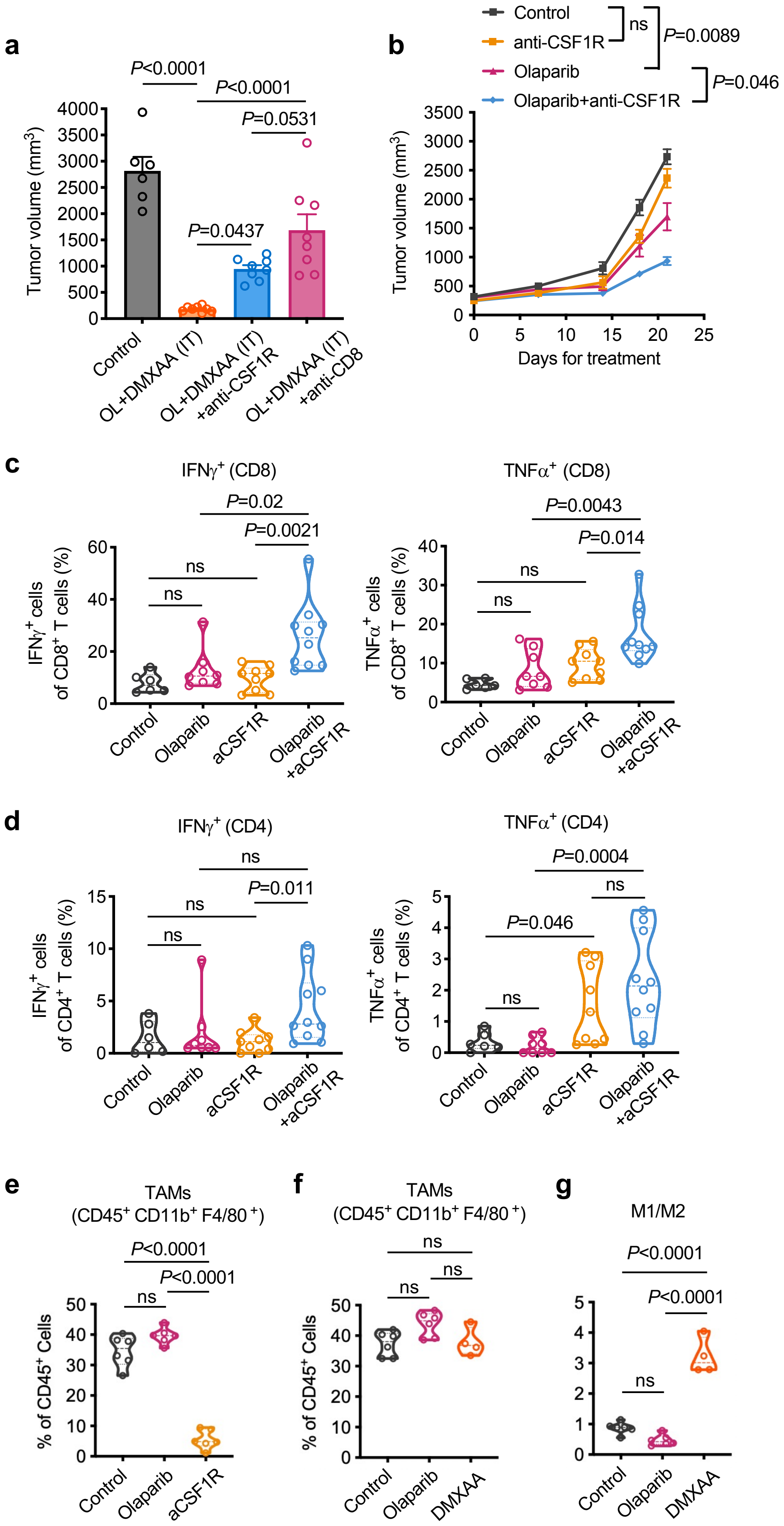

**Supplementary Fig. 6. Systemic delivery of a STING agonist potentiates the therapeutic efficacy of olaparib *in vivo*.**

**(a)** Tumor growth of BP-sgSTING allografts in FVB mice treated with intratumoral (IT) injections of DMXAA as a single agent or in combination with olaparib. Control, n = 4 tumors; DMXAA, n = 4 tumors; DMXAA + Olaparib, n = 4 tumors.

**(b)** Analysis of immune infiltration into BP and BP-sgSTING tumors in FVB mice before treatment. Each dot represents data from a single tumor. n = 6 tumors for each group.

**(c)** Analysis of intratumoral immune cells from BP-sgSTING tumors after a single IT or intraperitoneal (IP) injection of DMXAA and seven days of treatment with olaparib. Each dot represents data from a single tumor. n = 10 tumors for each group.

**(d)** Western blots for BRCA1 and VINCULIN in EO771-sgControl (Ctrl) and EO771-sgBRCA1 tumor cells. Representative blots of two independent experiments are shown.

**(e)** Clonogenic assay measuring the response of EO771-sgCtrl and EO771-sgBRCA1 cells to olaparib. Representative images are shown on the left, and quantification of crystal violet staining is shown on the right (n = 3 independent experiments).

**(f)** EO771-sgBRCA1 tumor growth in WT C57BL/6J mice (**left**) or STING-KO mice (**right**) treated with olaparib combined with IP injections of DMXAA. **Left**: Control, n = 9 tumors; olaparib + DMXAA = 10 tumors. **Right**: Control, n = 8 tumors; olaparib + DMXAA, n = 10 tumors.

**(g)** Western blots for STING and VINCULIN in EO771-sgBRCA1 and EO771-sgBRCA1-sgSTING cells. Representative blots of two independent experiments are shown.

**(h)** EO771-sgBRCA1-sgSTING tumor growth in WT C57BL/6J mice treated with olaparib combined with IP injections of DMXAA. Control, n = 10; olaparib + DMXAA, n = 10.

**(i)** Survival of EO771-sgBRCA1-sgSTING tumor-bearing WT C57BL/6J mice treated with olaparib combined with DMXAA via IT or IP injection. n = 5 mice for each group.

Data are presented as mean ± SEM (**a** and **e**), or median with quartiles (violin plots, **b** and **c**). Two-way analysis of variance (ANOVA) (**a**). Two-tailed unpaired t test (**b**). One-way ANOVA (**c** and **e**). Log-rank Mantel-Cox test (**i**). ns, not significant. Source data are provided as a Source Data file.

**Supplementary Fig. 6**

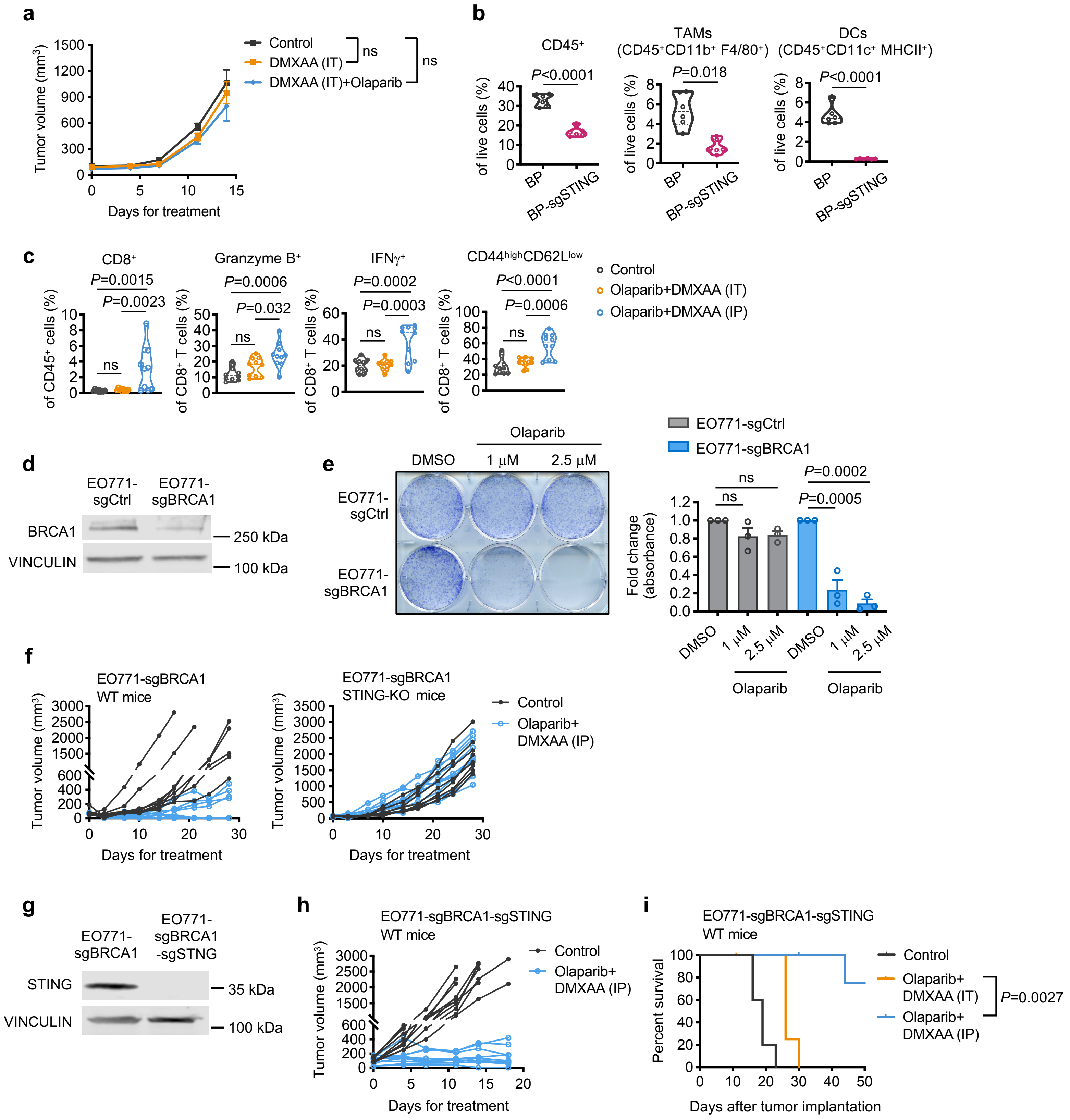

**Supplementary Fig. 7 Gating strategies for flow cytometry.**

**(a)** Gating strategies for TAM sorting described in Fig. 1e, 3g, and 4d.

**(b)** Gating strategies for flow cytometry analyses described in Fig. 1f, 1g and Supplementary Fig. 2k.

**(c)** Gating strategies for flow cytometry analyses described in Fig. 2b, 4c, 4d, 4f, 4g, 6c and Supplementary Fig. 2b, 2d, 2e, 2j.

**(d)** Gating strategies for flow cytometry analyses described in Supplementary Fig. 2c, 2g, 2i.

**(e)** Gating strategies for flow cytometry analyses described in Fig. 3c, 3e, 4i and Supplementary Fig. 3e and 3g.

**(e)** Gating strategies for flow cytometry analyses described in Supplementary Fig. 2c, 2g, 2i.

**(f)** Gating strategies for flow cytometry analyses described in Fig. 3f and Supplementary Fig. 3f, 3h.

**(g)** Gating strategies for flow cytometry analyses described in Fig. 3j.

**(h)** Gating strategies for flow cytometry analyses described in Fig. 4h.

**(i)** Gating strategies for flow cytometry analyses described in Supplementary Fig. 4c.

**(j)** Gating strategies for flow cytometry analyses described in Fig. 3i, 5c and Supplementary Fig. 5c, 5d, 5e, 5f, 5g, 6b, 6c.

All flow cytometry plot axes are displayed in logarithmic scale, except for forward side scatter (FSC) for which a linear scale was used.

**Supplementary Fig. 7**

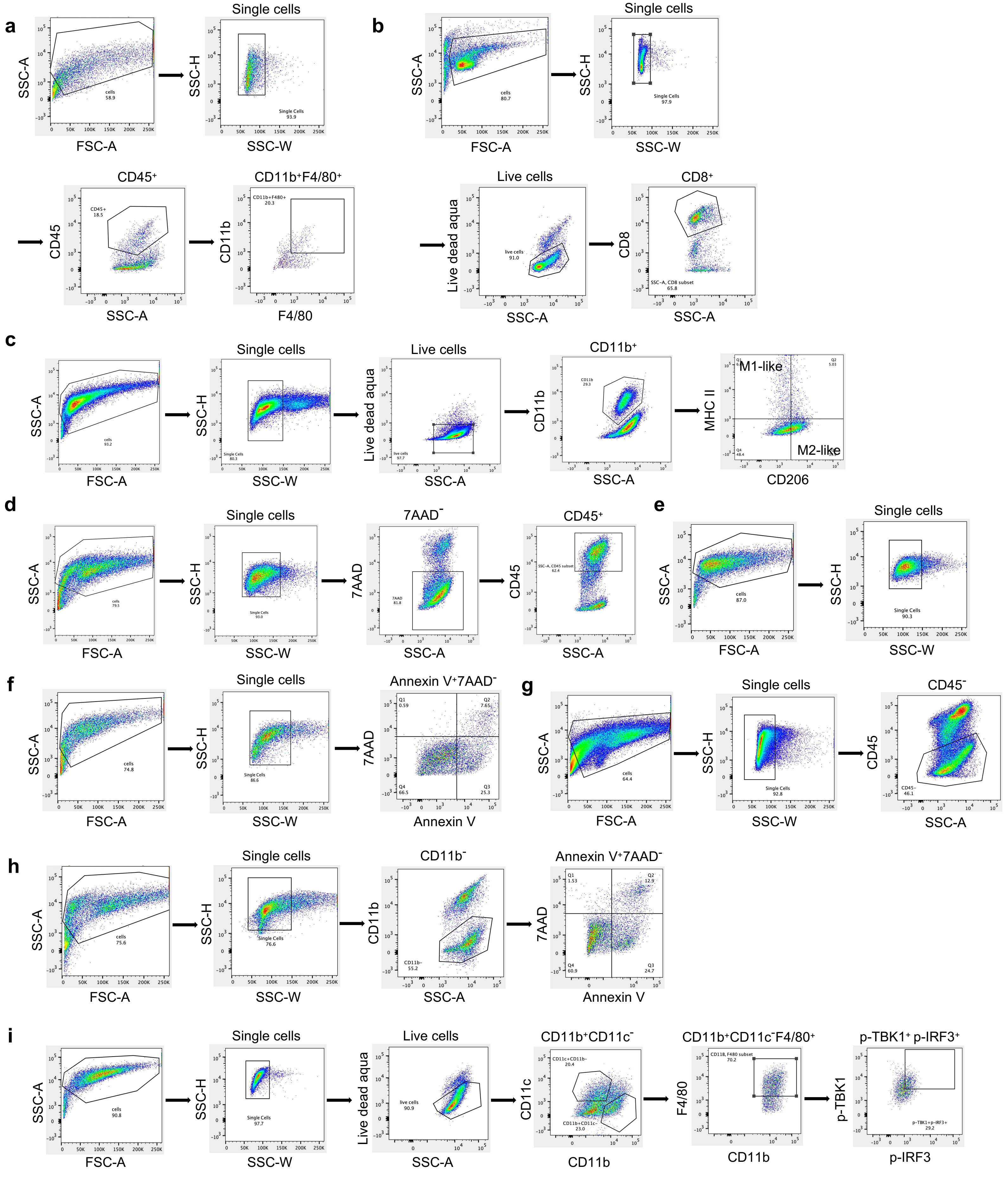

**Supplementary Fig. 7 (continued)**

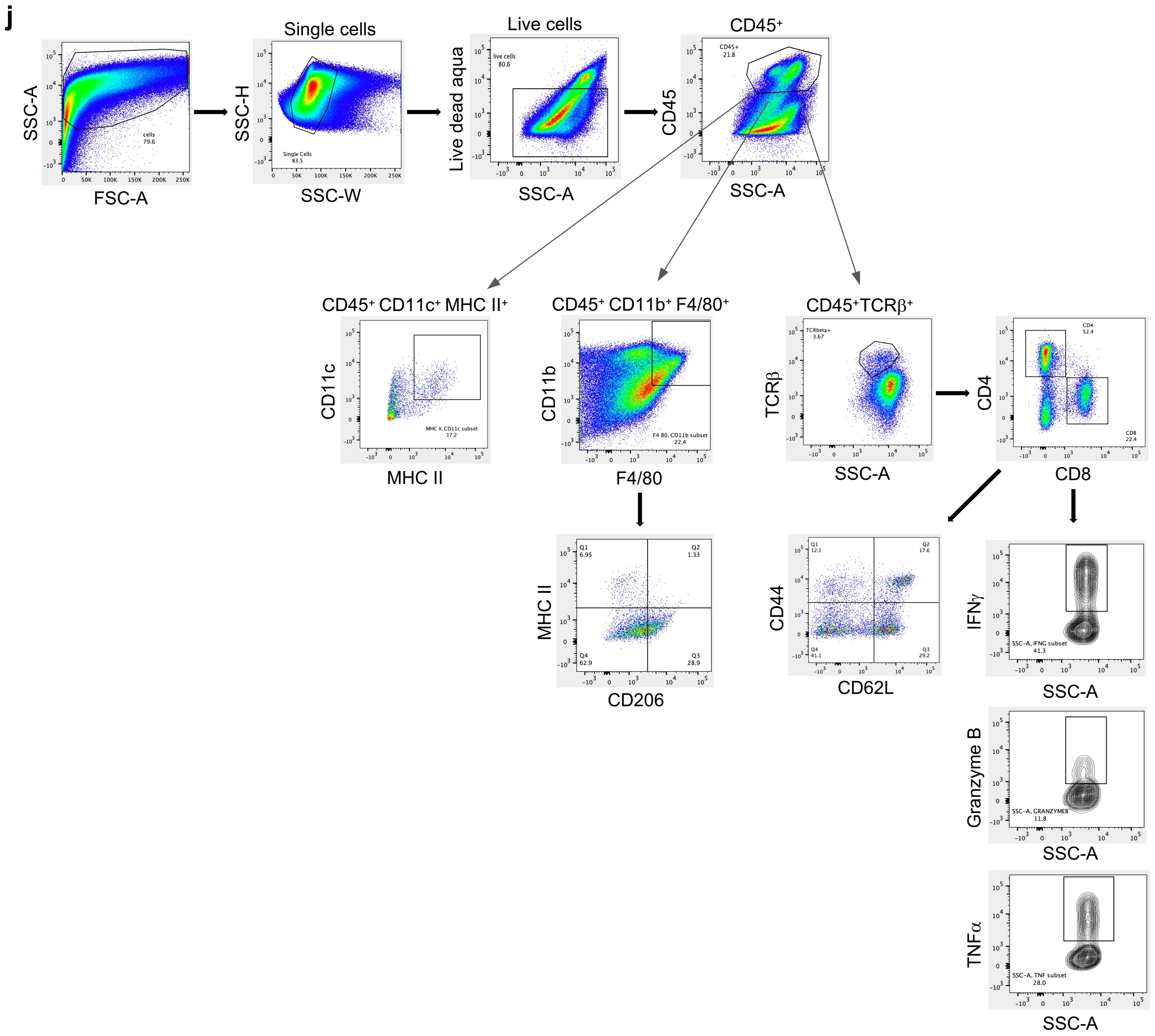

Supplement: Supplementary file 1 — Supplementary Information [file 41467_2022_30568_MOESM1_ESM.pdf]
