# Supplementary material for: STING agonism reprograms tumor-associated macrophages and overcomes resistance to PARP inhibition in BRCA1-deficient models of breast cancer
Source: Nat Commun. 2022 May 31;13:3022. doi: 10.1038/s41467-022-30568-1 (PMC9156717; doi:10.1038/s41467-022-30568-1)
Supplement: Supplementary file 2 — Reporting Summary [file 41467_2022_30568_MOESM2_ESM.pdf]

nature portfolio

Corresponding author(s): Jean J. Zhao

Last updated by author(s): Apr 24, 2022

Reporting Summary

Nature Portfolio wishes to improve the reproducibility of the work that we publish. This form provides structure for consistency and transparency in reporting. For further information on Nature Portfolio policies, see our Editorial Policies and the Editorial Policy Checklist.

Statistics

For all statistical analyses, confirm that the following items are present in the figure legend, table legend, main text, or Methods section.

n/a	Confirmed	
☐	☒	The exact sample size (*n*) for each experimental group/condition, given as a discrete number and unit of measurement
☐	☒	A statement on whether measurements were taken from distinct samples or whether the same sample was measured repeatedly
☐	☒	The statistical test(s) used AND whether they are one- or two-sided<br>*Only common tests should be described solely by name; describe more complex techniques in the Methods section.*
☒	☐	A description of all covariates tested
☐	☒	A description of any assumptions or corrections, such as tests of normality and adjustment for multiple comparisons
☐	☒	A full description of the statistical parameters including central tendency (e.g. means) or other basic estimates (e.g. regression coefficient) AND variation (e.g. standard deviation) or associated estimates of uncertainty (e.g. confidence intervals)
☐	☒	For null hypothesis testing, the test statistic (e.g. *F*, *t*, *r*) with confidence intervals, effect sizes, degrees of freedom and *P* value noted<br>*Give P values as exact values whenever suitable.*
☒	☐	For Bayesian analysis, information on the choice of priors and Markov chain Monte Carlo settings
☒	☐	For hierarchical and complex designs, identification of the appropriate level for tests and full reporting of outcomes
☒	☐	Estimates of effect sizes (e.g. Cohen's *d*, Pearson's *r*), indicating how they were calculated

Our web collection on statistics for biologists contains articles on many of the points above.

Software and code

Policy information about availability of computer code

Data collection	BD FACSDiva (version 6), Ion Torrent platform
Data analysis	GraphPad Prism (version 9.3.1), FlowJo (version 10.4), Image J, R software (version 3.6.3), R Bioconductor (version 3.10), R package 'affy' (version 1.64.0), R package 'Gene Set Variation Analysis (GSVA)' (version 1.34.0), R package 'ggplot2' (version 3.3.5) , R package 'ClusterProfiler' (version 3.14.3), R package 'gplots' (version 3.1.1) , DESeq2 package (version 1.26.0), Robust Multi-Array average (RMA) algorithm, GSEA (version 4.0.3), GSEAPreranked tool (v6.0.12), MSigDB v7.2

For manuscripts utilizing custom algorithms or software that are central to the research but not yet described in published literature, software must be made available to editors and reviewers. We strongly encourage code deposition in a community repository (e.g. GitHub). See the Nature Portfolio guidelines for submitting code & software for further information.

Data

Policy information about availability of data

All manuscripts must include a data availability statement. This statement should provide the following information, where applicable:

- Accession codes, unique identifiers, or web links for publicly available datasets
- A description of any restrictions on data availability
- For clinical datasets or third party data, please ensure that the statement adheres to our policy

Transcriptomic data generated in this study have been deposited in Gene Expression Omnibus (GEO) under accession number GSE201017 (https://www.ncbi.nlm.nih.gov/geo/query/acc.cgi?acc=GSE201017). Publicly available datasets used in this study are available in GEO under accession numbers GSE62944 (https://www.ncbi.nlm.nih.gov/geo/query/acc.cgi?acc=GSE62944), GSE63885 (https://www.ncbi.nlm.nih.gov/geo/query/acc.cgi?acc=GSE63885), and GSE27830 (https://www.ncbi.nlm.nih.gov/geo/query/acc.cgi?acc=GSE27830). BRCA1 mutation data for the TCGA cohort were downloaded from Riaz et al.. BRCA1 mutation

March 2021

data for the GSE63885 and GSE27830 cohorts were retrieved from the corresponding clinical data stored in the GEO database. The remaining data are available within the Article, Supplementary Information or Source Data file. Source data are provided with this paper.

Field-specific reporting

Please select the one below that is the best fit for your research. If you are not sure, read the appropriate sections before making your selection.

☒ Life sciences ☐ Behavioural & social sciences ☐ Ecological, evolutionary & environmental sciences

For a reference copy of the document with all sections, see nature.com/documents/nr-reporting-summary-flat.pdf

Life sciences study design

All studies must disclose on these points even when the disclosure is negative.

Sample size	For in vivo experiments, samples sizes were determined based on our previous experience with the models utilized, including experience in variability of tumor growth [Cell Rep. 2018 Dec 11;25(11):2972-2980.e5. Nature. 2017 Aug 24;548(7668):471-475.]. For the in vitro experiments, sample size calculation was not performed. The sample size was determined by the number of biological replicates required for measuring statistical significance, and the sample sizes were chosen to support meaningful conclusions based on our experience with the experiments we conducted [Cell Rep. 2018 Dec 11;25(11):2972-2980.e5. Nature. 2017 Aug 24;548(7668):471-475.].
Data exclusions	No data were excluded from analysis.
Replication	All experimental findings were replicated successfully. Numbers of attempts of replication are described in the Figure Legends.
Randomization	For in vivo experiments, age-matched mice were randomized into groups of equal average tumor volume prior to treatment initiation. For in vitro experiments, samples were allocated randomly for culture and treatments.
Blinding	For in vivo experiments, tumor measurements and downstream analyses of tissues (Immunohistochemistry and Flow cytometry) were performed in a blinded fashion as the researchers conducting these assays were not aware of treatment groups until data gathering was complete. Researchers were not fully blinded to the drug treatments as the drugs used in this study had different dosing regimens. For most of in vitro experiments, blinding was not performed as the individual who conducted the cell culture and treatments was also involved in sample collection and analysis. However, researchers who processed samples for RNA sequencing were blinded to the cell culture conditions and treatments for each group.

Reporting for specific materials, systems and methods

We require information from authors about some types of materials, experimental systems and methods used in many studies. Here, indicate whether each material, system or method listed is relevant to your study. If you are not sure if a list item applies to your research, read the appropriate section before selecting a response.

Materials & experimental systems

n/a	Involved in the study	
☐	☒	Antibodies
☐	☒	Eukaryotic cell lines
☒	☐	Palaeontology and archaeology
☐	☒	Animals and other organisms
☒	☐	Human research participants
☒	☐	Clinical data
☒	☐	Dual use research of concern

Methods

n/a	Involved in the study	
☒	☐	ChIP-seq
☐	☒	Flow cytometry
☒	☐	MRI-based neuroimaging

Antibodies

Antibodies used

Anti-mouse CD16/32 (clone 93), BioLegend, #101320, 1:50;
Human TruStain FcX (Fc Receptor Blocking Solution), BioLegend, #422302, 1:20;
FITC anti-mouse CD45 (clone 30-F11), BioLegend, #103108, 1:100;
FITC anti-human CD45 (clone HI30), BioLegend, #304006, 1:20;
PerCP/Cy5.5 anti-mouse TCR β chain (clone H57-597), BioLegend, #109228, 1:100;
PE/Dazzle 594 anti-mouse CD3ε (clone 145-2C11), BioLegend, #100348, 1:100;
APC/Cy7 anti-mouse CD4 (clone RM4-5), BioLegend, #100526, 1:100;
Alexa Fluor 700 anti-mouse CD8a (clone 53-6.7), BioLegend, #100730, 1:100;
Brilliant Violet 605 anti-mouse/human CD44 (clone IM7), BioLegend, #103047, 1:100;
Brilliant Violet 711 anti-mouse CD62L (clone MEL-14), BioLegend, #104445, 1:100;
PE/Dazzle 594 anti-mouse CD25 (clone PC61), BioLegend, #102048, 1:100;
PE anti-mouse IFN-γ (clone XMG1.2), BioLegend, #505808, 1:100;
APC anti-mouse TNF-α (clone MP6-XT22), BioLegend, #506308, 1:100;

PE/Cy7 anti-mouse Granzyme B (clone NGZB), eBioscience, # 25-8898-82, 1:100;
PE/Cy7 anti-mouse PD-1 (clone 29F.1A12), BioLegend, # 135216, 1:100;
PE/Cy7 anti-mouse CD11c (clone N418), BioLegend, #117318, 1:100;
APC/Cy7 anti-mouse I-A/I-E (clone M5/114.15.2), BioLegend, #107628, 1:100;
APC/Cy7 anti-human HLA-DR (clone L243), BioLegend, #307618, 1:20
Pacific Blue anti-mouse CD86 (clone GL-1), BioLegend, #105022, 1:100;
Brilliant Violet 650 anti-mouse/human CD11b (clone M1/70), BioLegend, #101259; 1:100
PerCP/Cy5.5 anti-mouse/human CD11b (clone M1/70), BioLegend, #101228, 1:100;
Alexa Fluor 488 anti-mouse/human CD11b (clone M1/70), BioLegend, #101219, 1:100;
APC anti-mouse F4/80 (clone BM8), BioLegend, # 123116, 1:100;
Brilliant Violet 605 anti-mouse F4/80 (clone BM8), BioLegend, # 123133, 1:100;
Brilliant Violet 711 anti-mouse CD206 (clone MMR), BioLegend, #141727, 1:100;
Alexa Fluor 700 anti-mouse CD206 (clone MMR), BioLegend, #141734, 1:100;
PE anti-human CD163 (clone GHI/61), BioLegend, #333606, 1:20;
PE anti-mouse/human phospho-TBK1 (Ser172) (clone D52C2), Cell Signaling Tech., #13498S, 1:50;
Alexa Fluor 647 anti-mouse/human phospho-IRF-3 (Ser396) (clone D6O1M), Cell Signaling Tech, #10327S, 1:50;
PE anti-H2A.X Phospho (Ser139) (clone 2F3), BioLegend, #613412, 1:20;
Alexa Fluor 488 Pan-Cytokeratin (clone C11) Alexa Fluor 488, Santa Cruz Biotechnology, SC-8018, 1:50;
Goat Anti-Mouse IgG Polyclonal Antibody DyLight 800, Rockland, #RL610-145-002, 1:2000;
Goat anti-Rabbit IgG (H+L) Highly Cross-Adsorbed Secondary Antibody, Alexa Fluor 680, Thermo Fisher, #A-21109, 1:2000;
Anti-double stranded DNA Antibody (anti-dsDNA), clone AE-2, Sigma, #MAB1293, 1:200;
Goat anti-Mouse IgG (H+L) Highly Cross-Adsorbed Secondary Antibody, Alexa Fluor 594, Thermo Fisher, #A-11032, 1:400;
InVivoMAb anti-mouse CSF1R neutralizing antibody (clone CD115), BioXcell, BE0213;
InVivoMAb anti-mouse CD8α neutralizing antibody (clone YTS 169.4), BioXcell, BE0117;
InVivoMAb anti-mouse IFNAR-1 neutralizing antibody (clone MAR1-5A3), BioXcell, BE0241;
InVivoMAb rat IgG2b isotype control, anti-keyhole limpet hemocyanin (clone LTF-2), BioXcell, BE0090;
Anti-PD-1 antibody (clone, 332.8H3) was generated in the laboratory of Dr. Gordon Freeman at Dana-Farber Cancer Institute [Cancer Immunol Res. 2016 Feb;4(2):124-35.];
BRCA1 antibody, Abcam, ab238983, 1:500;
ERalpha antibody (SP1), Fisher, RM9101S0, 1:200;
HER2 antibody (29D8), Cell Signaling Tech, 2165, 1:500;
Ki67 antibody, Abcam, ab15580, 1:500;
Pan-Cytokeratin (AE1/AE3), eBioscience, 53-9003-82, 1:100;
STING (D2P2F), Cell Signaling Tech, 13647S, 1:1000;
CD4 antibody (4SM95), eBioscience, 41-9766-82, 1:100;
CD8a antibody (4SM16), eBioscience, 14-0195-82, 1:300;
CD11b antibody (EPR1344), Abcam; ab204471, 1:100;
CD11c antibody (D1V9Y), Cell Signaling Tech, 97585S, 1:100;
F4/80 antibody (BM8), BioLegend, 123120, 1:100;
VINCULIN (hVIN-1), Sigma, V9131, 1:2000.

Validation

Anti-PD-1 antibody (clone, 332.8H3) was generated and validated in the laboratory of Dr. Gordon Freeman at Dana-Farber Cancer Institute [Cancer Immunol Res. 2016 Feb;4(2):124-35.].

All the other antibodies used in this study are commercially available and have been verified by the manufacturers according to the data on their websites.
Anti-mouse CD16/32 (clone 93), BioLegend, #101320
https://www.biolegend.com/en-us/products/trustain-fcx-anti-mouse-cd16-32-antibody-5683

Human TruStain FcX (Fc Receptor Blocking Solution), BioLegend, #422302
https://www.biolegend.com/en-us/products/human-trustain-fcx-fc-receptor-blocking-solution-6462

FITC anti-mouse CD45 (clone 30-F11), BioLegend, #103108
https://www.biolegend.com/en-us/products/fitc-anti-mouse-cd45-antibody-99

FITC anti-human CD45 (clone HI30), BioLegend, #304006
https://www.biolegend.com/en-us/products/fitc-anti-human-cd45-antibody-707

PerCP/Cy5.5 anti-mouse TCR β chain (clone H57-597), BioLegend, #109228
https://www.biolegend.com/en-us/products/percp-cyanine5-5-anti-mouse-tcr-beta-chain-antibody-5603

PE/Dazzle 594 anti-mouse CD3ε (clone 145-2C11), BioLegend, #100348
https://www.biolegend.com/en-us/products/pe-dazzle-594-anti-mouse-cd3epsilon-antibody-10066

APC/Cy7 anti-mouse CD4 (clone RM4-5), BioLegend, #100526
https://www.biolegend.com/en-us/products/apc-cyanine7-anti-mouse-cd4-antibody-1937

Alexa Fluor 700 anti-mouse CD8a (clone 53-6.7), BioLegend, #100730
https://www.biolegend.com/en-us/products/alexa-fluor-700-anti-mouse-cd8a-antibody-3387

Brilliant Violet 605 anti-mouse/human CD44 (clone IM7), BioLegend, #103047
https://www.biolegend.com/en-us/products/brilliant-violet-605-anti-mouse-human-cd44-antibody-8807

Brilliant Violet 711 anti-mouse CD62L (clone MEL-14), BioLegend, #104445
https://www.biolegend.com/en-us/products/brilliant-violet-711-anti-mouse-cd62l-antibody-10317

PE/Dazzle 594 anti-mouse CD25 (clone PC61), BioLegend, #102048
https://www.biolegend.com/en-us/products/pe-dazzle-594-anti-mouse-cd25-antibody-10220

PE anti-mouse IFN-γ (clone XMG1.2), BioLegend, #505808
https://www.biolegend.com/en-us/products/pe-anti-mouse-ifn-gamma-antibody-997

APC anti-mouse TNF-α (clone MP6-XT22), BioLegend, #506308
https://www.biolegend.com/en-us/products/apc-anti-mouse-tnf-alpha-antibody-975

PE/Cy7 anti-mouse Granzyme B (clone NGZB), eBioscience, # 25-8898-82
https://www.thermofisher.com/antibody/product/Granzyme-B-Antibody-clone-NGZB-Monoclonal/25-8898-82

PE/Cy7 anti-mouse PD-1 (clone 29F.1A12), BioLegend, # 135216
https://www.biolegend.com/en-us/products/pe-cyanine7-anti-mouse-cd279-pd-1-antibody-7005

PE/Cy7 anti-mouse CD11c (clone N418), BioLegend, #117318
https://www.biolegend.com/en-us/products/pe-cyanine7-anti-mouse-cd11c-antibody-3086

APC/Cy7 anti-mouse I-A/I-E (clone M5/114.15.2), BioLegend, #107628
https://www.biolegend.com/en-us/products/apc-cyanine7-anti-mouse-i-a-i-e-antibody-5966

APC/Cy7 anti-human HLA-DR (clone L243), BioLegend, #307618
https://www.biolegend.com/en-us/products/apc-cyanine7-anti-human-hla-dr-antibody-2863

Pacific Blue anti-mouse CD86 (clone GL-1), BioLegend, #105022
https://www.biolegend.com/en-us/products/pacific-blue-anti-mouse-cd86-antibody-3122

Brilliant Violet 650 anti-mouse/human CD11b (clone M1/70), BioLegend, #101259
https://www.biolegend.com/en-us/products/brilliant-violet-650-anti-mouse-human-cd11b-antibody-7638

PerCP/Cy5.5 anti-mouse/human CD11b (clone M1/70), BioLegend, #101228
https://www.biolegend.com/en-us/products/percp-cyanine5-5-anti-mouse-human-cd11b-antibody-4257

Alexa Fluor 488 anti-mouse/human CD11b (clone M1/70), BioLegend, #101219
https://www.biolegend.com/en-us/products/alexa-fluor-488-anti-mouse-human-cd11b-antibody-2700

APC anti-mouse F4/80 (clone BM8), BioLegend, # 123116
https://www.biolegend.com/en-us/products/apc-anti-mouse-f4-80-antibody-4071

Brilliant Violet 605 anti-mouse F4/80 (clone BM8), BioLegend, # 123133
https://www.biolegend.com/en-us/products/brilliant-violet-605-anti-mouse-f4-80-antibody-8702

Brilliant Violet 711 anti-mouse CD206 (clone MMR), BioLegend, #141727
https://www.biolegend.com/en-us/products/brilliant-violet-711-anti-mouse-cd206-mmr-antibody-12012

Alexa Fluor 700 anti-mouse CD206 (clone MMR), BioLegend, #141734
https://www.biolegend.com/en-us/products/alexa-fluor-700-anti-mouse-cd206-mmr-antibody-13456

PE anti-human CD163 (clone GHI/61), BioLegend, #333606
https://www.biolegend.com/en-us/products/pe-anti-human-cd163-antibody-4793

PE anti-mouse/human phospho-TBK1 (Ser172) (clone D52C2), Cell Signaling Tech., #13498S
https://www.cellsignal.com/products/antibody-conjugates/phospho-tbk1-nak-ser172-d52c2-xp-rabbit-mab-pe-conjugate/13498?site-search-type=Products&N=4294956287&Ntt=13498s&fromPage=plp&_requestid=6330744

Alexa Fluor 647 anti-mouse/human phospho-IRF-3 (Ser396) (clone D6O1M), Cell Signaling Tech, #10327S
https://www.cellsignal.com/products/antibody-conjugates/phospho-irf-3-ser396-d6o1m-rabbit-mab-alexa-fluor-647-conjugate/10327?site-search-type=Products&N=4294956287&Ntt=10327s&fromPage=plp&_requestid=6330788

PE anti-H2A.X Phospho (Ser139) (clone 2F3), BioLegend, #613412
https://www.biolegend.com/en-us/products/pe-anti-h2a-x-phospho-ser139-antibody-13292

Alexa Fluor 488 Pan-Cytokeratin (clone C11) Alexa Fluor 488, Santa Cruz Biotechnology, SC-8018
https://www.scbt.com/p/pan-cytokeratin-antibody-c11

Goat Anti-Mouse IgG Polyclonal Antibody DyLight 800, Rockland, #RL610-145-002
https://www.rockland.com/categories/secondary-antibodies/mouse-igg-hl-antibody-dylight-800-conjugated-610-145-002/

Goat anti-Rabbit IgG (H+L) Highly Cross-Adsorbed Secondary Antibody, Alexa Fluor 680, Thermo Fisher, #A-21109
https://www.thermofisher.com/antibody/product/Goat-anti-Rabbit-IgG-H-L-Highly-Cross-Adsorbed-Secondary-Antibody-Polyclonal/A-21109

Anti-double stranded DNA Antibody (anti-dsDNA), clone AE-2, Sigma, #MAB1293
https://www.sigmaaldrich.com/US/en/product/mm/mab1293?gclid=CjwKCAjwx46TBhBhEiwArA_DjJ71oY2taiXEVYukQDZEDi_qqtXfjAhADFBsEqMAaNbDzs_XbEFb4xoC8_cQAvD_BwE

Goat anti-Mouse IgG (H+L) Highly Cross-Adsorbed Secondary Antibody, Alexa Fluor 594, Thermo Fisher, #A-11032
https://www.thermofisher.com/antibody/product/Goat-anti-Mouse-IgG-H-L-Highly-Cross-Adsorbed-Secondary-Antibody-Polyclonal/A-11032

InVivoMAb anti-mouse CSF1R neutralizing antibody (clone CD115), BioXcell, BE0213
https://bxcell.com/product/anti-cd115-anti-csf-1/

InVivoMAb anti-mouse CD8α neutralizing antibody (clone YTS 169.4), BioXcell, BE0117
https://bxcell.com/product/m-cd8/

InVivoMAb anti-mouse IFNAR-1 neutralizing antibody (clone MAR1-5A3), BioXcell, BE0241
https://bxcell.com/product/anti-m-ifnar-1/

InVivoMAb rat IgG2b isotype control, anti-keyhole limpet hemocyanin (clone LTF-2), BioXcell, BE0090
https://bxcell.com/product/rat-igg2b-isotype-control/

Anti-PD-1 antibody (clone, 332.8H3) was generated in the laboratory of Dr. Gordon Freeman at Dana-Farber Cancer Institute [Cancer Immunol Res. 2016 Feb;4(2):124-35.]

BRCA1 antibody, Abcam, ab238983, 1:500
https://www.abcam.com/brca1-antibody-ab238983.html

ERalpha antibody (SP1), Fisher, RM9101S0, 1:200
https://www.fishersci.com/shop/products/lab-vision-estrogen-receptor-sp1-rabbit-monoclonal-antibody-fitc-bsa-azide/RM9101S0?searchHijack=true&searchTerm=RM9101S0&searchType=RAPID&matchedCatNo=RM9101S0

HER2 antibody (29D8), Cell Signaling Tech, 2165, 1:500
https://www.cellsignal.com/products/primary-antibodies/her2-erbb2-29d8-rabbit-mab/2165

Ki67 antibody, Abcam, ab15580, 1:500
https://www.abcam.com/ki67-antibody-ab15580.html

Pan-Cytokeratin (AE1/AE3), eBioscience, 53-9003-82
https://www.thermofisher.com/antibody/product/Pan-Cytokeratin-Antibody-clone-AE1-AE3-Monoclonal/53-9003-82

STING (D2P2F), Cell Signaling Tech, 13647S
https://www.cellsignal.com/products/primary-antibodies/sting-d2p2f-rabbit-mab/13647?site-search-type=Products&N=4294956287&Ntt=13647s&fromPage=plp&_requestid=6332437

CD4 antibody (4SM95), eBioscience, 41-9766-82
https://www.thermofisher.com/antibody/product/CD4-Antibody-clone-4SM95-Monoclonal/41-9766-82

CD8a antibody (4SM16), eBioscience, 14-0195-82
https://www.thermofisher.com/antibody/product/CD8a-Antibody-clone-4SM16-Monoclonal/14-0195-82

CD11b antibody (EPR1344), Abcam; ab204471
https://www.abcam.com/cd11b-antibody-epr1344-alexa-fluor-647-ab204471.html

CD11c antibody (D1V9Y), Cell Signaling Tech, 97585S
https://www.cellsignal.com/products/primary-antibodies/cd11c-d1v9y-rabbit-mab/97585?site-search-type=Products&N=4294956287&Ntt=97585s&fromPage=plp&_requestid=6332652

F4/80 antibody (BM8), BioLegend, 123120
https://www.biolegend.com/en-us/products/alexa-fluor-488-anti-mouse-f4-80-antibody-4073

VINCULIN (hVIN-1), Sigma, V9131
https://www.sigmaaldrich.com/US/en/product/sigma/v9131

Eukaryotic cell lines

Policy information about cell lines

Cell line source(s)	EO771 cell line (# 940001) was purchased from CH3 BioSystems. MDA-MB-436 (# HTB-130), HCC1937 (# CRL-2336), and THP-1 (# TIB-202) cell lines were obtained from ATCC.
Authentication	All cell lines were authenticated by short tandem repeat analysis.
Mycoplasma contamination	All cell lines tested negative for mycoplasma.
Commonly misidentified lines (See ICLAC register)	No commonly misidentified cell lines were used.

Animals and other organisms

Policy information about studies involving animals; ARRIVE guidelines recommended for reporting animal research

Laboratory animals	6 to 8-week-old female C57BL/6J (The Jackson Laboratory, # 000664) or female STING knockout mice (C57BL/6J-Tmem173gt/J, The Jackson Laboratory, # 017537) were used in the study for orthotopic injection of mammary tumor cells. Brca1loxP/loxP mouse line was kindly provided by Dr. Jos Jonkers's laboratory at Netherlands Cancer Institute. Trp53loxP/loxP mouse line was obtained from National Cancer Institute Mouse Repository (FVB.129P2-Trp53tm1Brn/Nci, # 01XC2). Brca1loxP/loxP and Trp53loxP/loxP mouse lines were both backcrossed into the FVB/N background for more than ten generations. To develop syngeneic genetically engineered mouse models (GEMMs) of Brca1-deficient breast cancer, Brca1loxP/loxP mice were further crossed with Trp53loxP/loxP mice. The resulting 8-12 weeks old Brca1loxP/loxP; Trp53loxP/loxP female mice were injected intraductally with adenovirus expressing Cre recombinase under a CMV promoter, which led to the development of mammary tumors driven by concurrent loss of Brca1 and Trp53. To evaluate the antitumor efficacy of treatments, mammary tumor cells for orthotopic injection were resuspended in serum-free DMEM containing 40% matrigel (Corning) and injected orthotopically into the mammary fat pads of 6 to 8-week-old female FVB/N mice (The Jackson Laboratory, # 001800).<br>Mice were housed in groups of no more than 5 per cage in standard closed plastic cages containing bedding, enrichment, food, and water, at controlled stable room temperature (64 to 79 °F) and humidity (30% to 70%), light/dark cycle 12 hours per day.
Wild animals	No wild animals were used in this study.
Field-collected samples	No field collections were used in this study.
Ethics oversight	All the animal experiments described in this study were performed according to animal protocols approved by the DFCI Institutional Animal Care and Use Committee (IACUC).

Note that full information on the approval of the study protocol must also be provided in the manuscript.

Flow Cytometry

Plots

Confirm that:

☒ The axis labels state the marker and fluorochrome used (e.g. CD4-FITC).

☒ The axis scales are clearly visible. Include numbers along axes only for bottom left plot of group (a 'group' is an analysis of identical markers).

☒ All plots are contour plots with outliers or pseudocolor plots.

☒ A numerical value for number of cells or percentage (with statistics) is provided.

Methodology

Sample preparation	To obtain single-cell suspensions, tumors were excised, minced and dissociated in collagenase/hyaluronidase buffer [DMEM with 5% FBS, 10 mM HEPES (Gibco), 100 μg/mL penicillin–streptomycin, 20 μg/mL DNase I (StemCell) and 1X collagenase/hyaluronidase (StemCell)] for 45 min at 37°C with agitation, followed by treatment with ammonium-chloride-potassium (ACK) buffer (Lonza) for red blood cell (RBC) lysis, and strained through a 70 μm strainer to remove undigested tumor tissues.
Instrument	LSRFortessa HTS (BD) and FACSAria-II SORP (BD) were used.
Software	FACSDiva software (BD) was used to collect the data, and the data was analyzed using FlowJo (BD).
Cell population abundance	After sorting, cells were counted and an equal number was prepared for each sample.
Gating strategy	Gating strategies are included in the Supplementary Information. Gates were set after appropriate compensation by single color stains.

☒ Tick this box to confirm that a figure exemplifying the gating strategy is provided in the Supplementary Information.
